# Catalytically Active Recombinant Cysteine Proteases of *Haemonchus contortus:* Their Ability to Degrade Host Blood Proteins and Modulate Coagulation

**DOI:** 10.3390/ijms262412077

**Published:** 2025-12-16

**Authors:** Athira C. Karunakaran, Mariam Bakshi, Arunraj M. Rajendrakumar, Jennifer H. Wilson-Welder, Raffi V. Aroian, Erich M. Schwarz, E. Jane Homan, Gary R. Ostroff, Ethiopia Beshah, Eliseo Miramontes, Marianne Dias Papadopoulos, Scott A. Bowdridge, Dante S. Zarlenga, Xiaoping Zhu, Wenbin Tuo

**Affiliations:** 1 Animal Parasitic Diseases Laboratory, Agricultural Research Service, U.S. Department of Agriculture, Beltsville, MD 20705, USA; 2 Oak Ridge Institute for Science and Education, Oak Ridge, TN 37830, USA; 3 Department of Veterinary Medicine, University of Maryland, College Park, MD 20740, USA; 4 Infectious Bacterial Diseases Research Unit, National Animal Disease Center, Agricultural Research Service, U.S. Department of Agriculture, Ames, IA 50010, USA; 5 Program in Molecular Medicine, University of Massachusetts Chan Medical School, Worcester, MA 01655, USA; 6 Department of Molecular Biology and Genetics, Cornell University, Ithaca, NY 14853, USA; 7 ioGenetics LLC, Madison, WI 53704, USA; 8 Davis College of Agriculture and Natural Resources, Morgantown, WV 26506, USA

**Keywords:** gastrointestinal nematode, *Haemonchus contortus*, cathepsin B, cysteine proteases, fibrinogen, blood clotting, recalcification time assay

## Abstract

*Haemonchus contortus* is a blood-feeding gastrointestinal nematode that significantly impacts the health and productivity of small ruminants. The burden of parasitism and the escalating incidence of anthelmintic resistance necessitate alternative control methods. Here, we characterize the enzymatic activities of five mammalian cell-expressed recombinant *H. contortus* cysteine proteases (HcCPs), which include two cathepsin B-like proteins (HcCBP1 and HcCBP2) and three cysteine protease 1 proteins (HcCP1a, HcCP1b, and HcCP1c). We hypothesize that these enzymes degrade host blood proteins, thereby facilitating the parasite’s nutrient acquisition and survival. Using synthetic cathepsin (cat) substrates, we show that HcCBP2 was the only protein that exhibited high catB/L but low catB or catK activity, which was inhibited by the cysteine protease inhibitor E-64. All mHcCPs degraded fibrinogen (Fg), which led to delayed plasma clotting, reduced clot density, and lysed plasma clots. All HcCPs partially degraded hemoglobin (Hb), except for mHcCBP2, which degraded Hb and bovine serum albumin completely and bovine IgG partially in the presence of a reducing agent. In conclusion, by sustaining blood feeding and facilitating immune evasion and nutrient acquisition, the HcCPs may play an essential role in the parasite’s survival. Thus, vaccines or cysteine protease inhibitors targeting these parasitic enzymes may mitigate or prevent infections.

## 1. Introduction

*Haemonchus contortus,* also referred to as the barber’s pole worm or wireworm, is a hematophagous gastrointestinal nematode parasite that inflicts significant harm upon small ruminants, mainly sheep and goats, and New World camelids [1,2]. The gravity of parasitism, persistent emergence, and widespread anthelmintic resistance necessitate an alternative control mechanism, such as a protective and cost-effective vaccine [3,4]. To develop effective and sustainable control strategies by identifying novel vaccine candidates, it is essential to understand the interaction between the parasite and its host. The life cycle of *H. contortus* includes both free-living and parasitic stages. The parasitic phase begins when animals ingest third-stage larvae (L3) while grazing. The larvae migrate to the abomasum, where they develop into fourth-stage larvae (L4) and then progress to the immature adult L5 stage. Upon reaching the L5 stage, the worms mature into dioecious adult worms. During development, they form a lancet in their buccal cavity. Using this lancet, they cut into the abomasal tissue, causing hemorrhages from which they feed on the leaking blood [5]. If conditions are unfavorable, the L4 enters a stage of developmental arrest or diapause, also known as hypobiosis [6,7,8].

The blood-feeding by *H. contortus* results in significant blood loss because a single adult worm can ingest approximately 30–50 μL of blood per day. Research indicates that sheep infested with 3000 to 4000 worms typically experience a daily blood loss of 150 to 200 milliliters [5,8,9]. To sustain this lifestyle, the parasite relies on its secretory enzymes, including cysteine proteases, to fulfill its needs in digestive and anticoagulant activities. These enzymes have great potential as vaccine candidates, as vaccination with gut extracts enriched for cysteine protease activities has shown up to 87% reduction in worm burden and a 93% reduction in egg output in sheep [10]. Another study investigated the immunoprotective effects of native cysteine protease-enriched fractions from adult *H. contortus* worms in caprine haemonchosis. These proteases exhibited optimal activity at pH 5, which was inhibited by the cysteine protease-specific inhibitor E-64 [11]. Immunizing goats with these fractions resulted in an 89% decrease in egg counts and a 68% decrease in worm counts compared to the control group. These findings suggest that cysteine proteases can be effective vaccine candidates against haemonchosis [11]. The protection is thought to be due to the host antibodies ingested by the parasite during blood feeding which disrupt the function mediated by the parasitic proteases.

In this study, we focused on identifying, expressing, and characterizing cysteine proteases (CPs) from *H. contortus.* These include two predicted cathepsin (cat) B-like proteins (HcCBP1 and HcCBP2) [12] and three cysteine protease 1 proteins (HcCP1a, HcCP1b, and HcCP1c) [13]. The HcCPs were produced using both bacterial and mammalian expression systems. We tested their ability to degrade host blood proteins under different pH and temperature conditions. Additionally, we performed cathepsin assays with synthetic substrates to evaluate HcCP activity against cathepsins (cat) B, B/L, and K. We also examined the effects of these proteases on blood coagulation through recalcification assay. Our results show that the recombinant HcCPs expressed in mammalian cells demonstrate enzymatic activity in degrading host blood proteins and regulating host blood coagulation through their fibrinogenolytic (HcCBP1 and 2, HcCP1a, b, and c), anticoagulant (HcCBP1, HcCP1a, b, and c), procoagulant (HcCBP2), and profibrinolytic (HcCP1a and HcCP1b) functions. Importantly, all HcCPs are present in *H. contortus*, and all HcCPs triggered an antibody response in sheep naturally infected with *H. contortus*, thereby underscoring their immunogenic properties. However, since HcCPs are secreted into the gastrointestinal tract of the parasite to facilitate nutrient absorption during the blood feeding process, the level of antigen exposure to the host, and, consequently, the host’s antibody production during natural infections, is inadequate to achieve effective parasite elimination. Therefore, HcCPs possess the potential to be developed as vaccine candidates for combating this infection.

## 2. Results

### 2.1. HcCP Sequence Analysis

Protein sequence alignment revealed that all five proteins share a sequence identity of 46–66%, where HcCP1a and HcCP1b have the highest sequence identity of 66%. Each protein has a signal peptide, and the proteins possess 14 conserved cysteine residues (Supplementary Figure S1). In addition, we performed sequence alignment of HcCPs with *Homo sapiens* catB and L, as well as with catB and L from *Ancylostoma caninum* and show that HcCPs exhibit a greater alignment with catBs from both *A. caninum* and *H. sapiens* than catLs from the same species (Supplementary Figure S2). Moreover, HcCBP1, HcCBP2 and HcCP1c share the His-His motif with human catB in the occluding loop which is characteristic of the exopeptidase sequence of human catB (Supplementary Figure S3) [14].

### 2.2. HcCPs Expressed in E. coli and FreeStyle™ 293-F Cells

**Figure 1:**
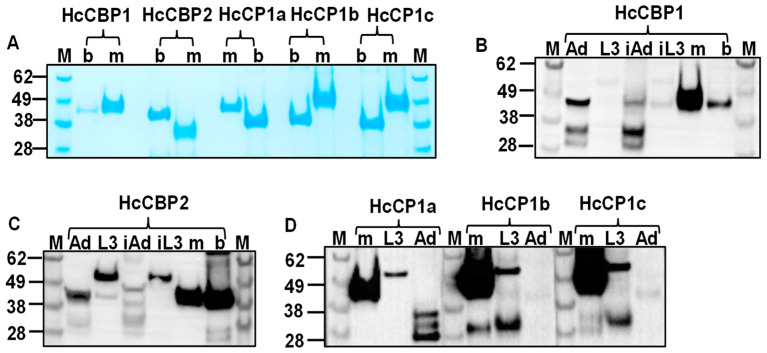
SDS-PAGE and Western blot analyses of native and recombinant *H. contortus* cysteine proteases (HcCPs) expressed in *E. coli* and FreeStyle 293-F cells. (**A**). Coomassie blue staining. Lane M, protein molecular standards; Lane b, *E*. *coli*-expressed recombinant HcCPs; Lane m, mammalian HcCPs. (**B**–**D**): Western blot analyses of native HcCBP1 (**B**), HcCBP2 (**C**), and HcCP1a/HcCP1b/HcCP1c (**D**) in *H. contortus* L3 and adult worm crude extract using antibodies specific to each of the HcCPs. Lanes Ad (soluble) and iAd (insoluble), *H. contortus* adult worm extract; Lanes L3 (soluble) and iL3 (insoluble), *H. contortus* L3 crude extract.

The HcCPs were expressed in *E. coli* (bHcCPs) and FreeStyle™ 293-F cells (mHcCPs) and purified using affinity chromatography. The purity of the proteins was evaluated by SDS-PAGE and Western blotting (Figure 1A; Supplementary Figure S4A,B). Each of the purified HcCPs migrated as a single band consistent with the predicted molecular mass. All bHcCPs had lower molecular weights compared to mHcCPs, except for bHcCBP2, which was slightly larger than the mammalian version (Figure 1A). The lower molecular weight of mHcCBP2 was caused by auto- and/or self-cleavage (Supplementary Figure S5).

### 2.3. The Presence of HcCPs in H. contortus L3 and Adults

Western blotting was performed to detect native HcCPs in lysates of both L3 and adult *H. contortus* using antisera raised against purified recombinant HcCPs (Figure 1B–D). The results showed that HcCBP1 was expressed at the adult stage in soluble and insoluble fractions but not in L3 (Figure 1B). For HcCBP2, it was expressed in both L3 and adult stages in soluble and insoluble fractions; however, it was present in both soluble and insoluble fractions of L3 extract with a higher molecular weight (Figure 1C). HcCP1a, b, and c were detectable in the soluble fraction of L3 extract (Figure 1D). HcCP1a was detected in the adult extract, but with a much lower molecular weight. HcCP1b and c were not detectable in the adult extract (Figure 1D).

### 2.4. Protease Activities of mHcCPs on Fg, oHb, IgG, or BSA

**Figure 2:**
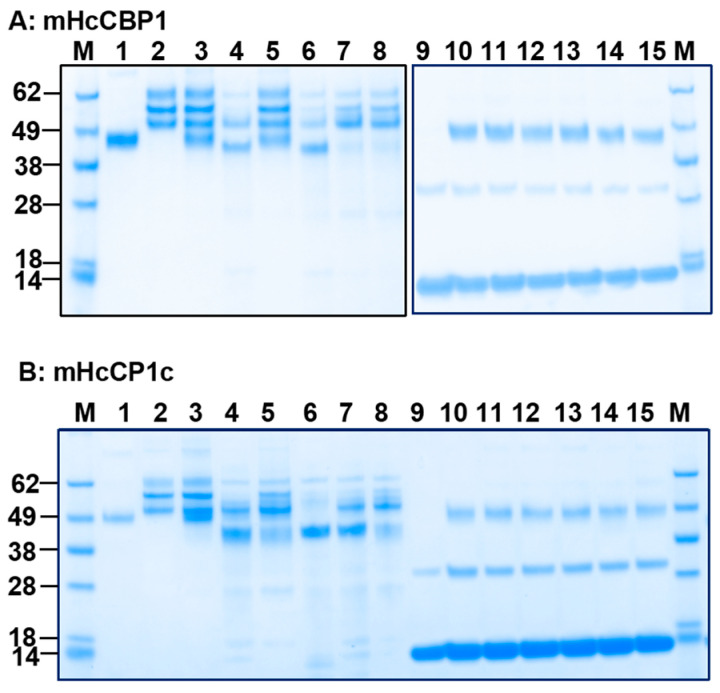
Representative results of recombinant *H. contortus* cysteine protease (HcCPs) activity on ovine fibrinogen (oFg; lanes 3–8) and ovine hemoglobin (oHb; lanes 10–15) at pH 4.0–8.0 at 37 °C for 16 h. Proteins were analyzed by SDS-PAGE followed by Coomassie blue staining. (**A**), mHcCBP1; (**B**), mHcCP1c; Lanes 3 and 10, 0.1 M acetate buffer (pH 4.0); Lanes 4 and 11, 0.1 M acetate buffer (pH 5.5); Lanes 5 and 12, 0.1 M phosphate buffer (pH 6.5); Lanes 6 and 13, PBS (pH 7.4); Lanes 7 and 14, 0.02 M Tris buffer (pH 8.0); and Lanes 8 and 15, cathepsin B assay buffer (pH 7–8). Lane M, protein standards. Lane 1, HcCPs alone; Lane 2, oFg alone; Lanes 3 through 8, oFg plus HcCPs, Lane 9, oHb alone; Lanes 10 through 15, oHb plus HcCPs.

**Figure 3:**
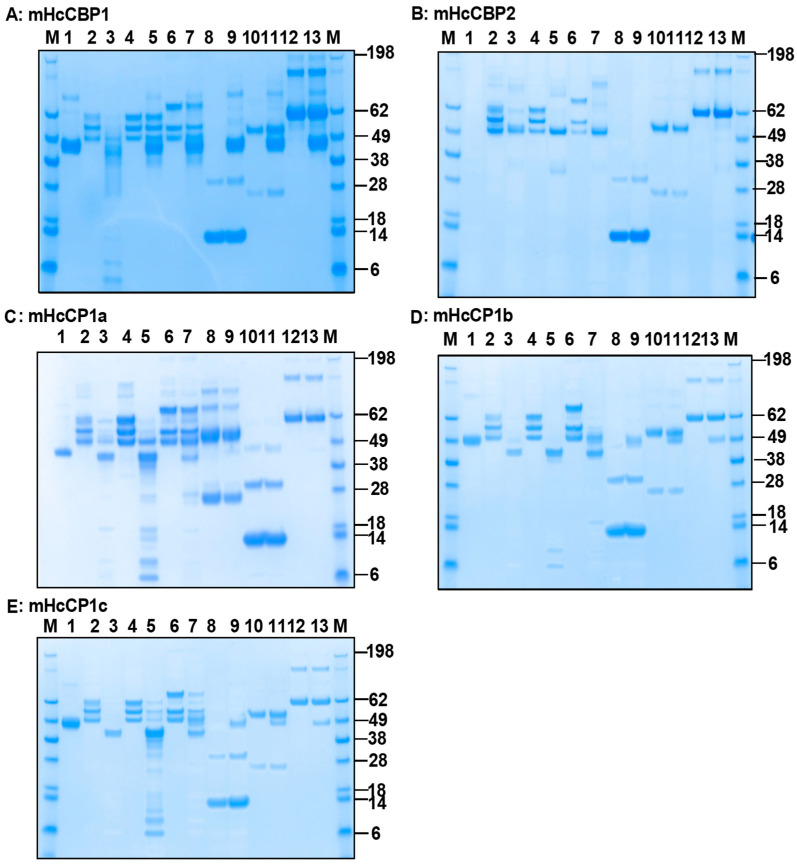
SDS-PAGE analysis of blood protein substrates digested by FreeStyle 293-F cells-expressed *H. contortus* cysteine proteases (mHcCPs), which include mHcCBP1 (**A**), mHcCBP2 (**B**), mHcCP1b (**D**), and mHcCP1c (**E**). Digestion was performed for 16 h at 37 °C in PBS, pH 7.4. Lane M, protein molecular weight standards; Lane 1, mHcCP; Lane 2, ovine fibrinogen (oFg); Lane 3, oFg and mHcCP; Lane 4, bovine fibrinogen (bFg); Lane 5, bFg and mHcCP; Lane 6, porcine fibrinogen (pFg); Lane 7, pFg and mHcCP; Lane 8, ovine hemoglobin (oHb), Lane 9, oHb and mHcCP; Lane 10, bovine immunoglobin G (bIgG); Lane 11, bIgG and mHcCP; Lane 12, bovine serum albumin (BSA); Lane 13, BSA and mHcCP. mHcCP1a (**C**), Lane M, protein molecular weight standards; Lane 1, mHcCP1a; Lane 2, oFg; Lane 3, oFg and mHcCP1a; Lane 4, bFg; Lane 5, bFg and mHcCP1a; Lane 6, pFg; Lane 7, pFg and mHcCP1a; Lane 8, bIgG; Lane 9, bIgG and mHcCP1a; Lane 10, oHb; Lane 11, oHb and mHcCP1a; Lane 12, BSA; Lane 13, BSA and mHcCP1a. The α, β, and ꎳ are three pairs of non-identical peptide chains of fibrinogen.

We evaluated the protease activity of bHcCPs across a pH range from 4.0 to 8.0. No enzymatic activity was demonstrated on host blood protein substrates such as ovine fibrinogen (oFg) or hemoglobin (oHb) (Supplementary Figure S6A–C). mHcCBP1 degraded oFg, but not oHb, under different conditions, including acetate buffer (pH 5.5), cathepsin assay buffer, PBS (pH 7.4), and Tris buffer (pH 8.0). mHcCBP1 was not active in acetate buffer at pH 4.0 or phosphate buffer at pH 6.5 and was only moderately active in degrading oFg in cathepsin assay buffer or at pH 8.0. It exhibited maximum activities degrading oFg at pH 5.5 and pH 7.4 (Figure 2A) but showed no activity on oHb at pH 4.0, 5.0, and 6.0 in the presence of DTT (Supplementary Figure S7). mHcCP1c was active in degrading oFg at various pH conditions; its maximum activity was detected at pH 5.5 and 7.4 (Figure 2B). Moreover, in PBS at pH 7.4, all mHcCPs were able to degrade Fg from sheep, cattle, and swine, but not oHb, IgG, and BSA (Figure 3A–E). mHcCBP1 almost completely degraded oFg yet had no detectable degradation on bovine and porcine Fgs (Figure 3A). mHcCBP2, however, degraded the alpha and beta chains of all 3 Fgs or reduced the alpha and beta chains to the size of the gamma chain (Figure 3B), and mHcCP1a, b and c were almost completely degraded ovine and bovine Fgs, but only partially degrading the porcine Fg (Figure 3C–E).

**Figure 4:**
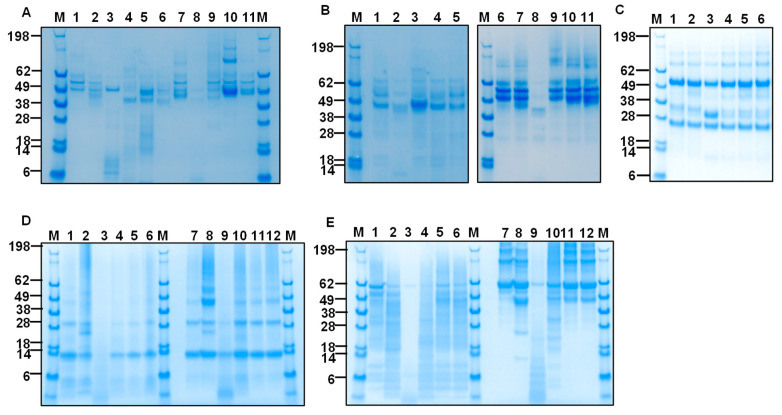
The ability of FreeStyle 293-F cells-expressed *H. contortus* cysteine proteases (mHcCPs) to degrade ovine fibrinogen (oFg), ovine hemoglobin (oHb), bovine immunoglobin G (bIgG), and bovine serum albumin (BSA) at 37 °C (**A**–**E**) pH 7.4 or 5.5 for 16 h. Proteins were analyzed by SDS-PAGE followed by Coomassie blue staining. (**A**), Lanes 1 through 6, pH 7.4 (PBS) without DTT; Lanes 7 through 11, pH 7.4 (PBS) with 10 mM DTT; Lane 1, oFg alone; Lanes 2 and 7, oFg and mHcCBP1; Lanes 3 and 8, oFg and mHcCBP2; Lanes 4 and 9, oFg and mHcCP1a; Lanes 5 and 10, oFg and mHcCP1b; Lanes 6 and 11, oFg and mHcCP1c. (**B**), Lanes 1 through 5, 20 mM NaCl in 10 mM Tris, pH 5.5, without DTT; Lanes 6 through 11, 20 mM NaCl and 10 mM DTT in 10 mM Tris. Lane 6, oFg alone; Lanes 1 and 7, oFg and mHcCBP1; Lanes 2 and 8, oFg and mHcCBP2; Lanes 3 and 9, oFg and mHcCP1a; Lanes 4 and 10, oFg and mHcCP1b; Lanes 5 and 11, oFg and mHcCP1c. Lane M, protein molecular weight standards. (**C**), Lane M, protein molecular weight standards; Lane 1, bIgG alone; Lanes 2 through 6, bIgG with mHcCPs which include mHcCBP1 (Lane2), mHcCBP2 (Lane 3), mHcCP1a (Lane 4), mHcCP1b (Lane 5), and mHcCP1c (Lane 6), Digestion was performed for 16 h at 37 °C in 20 mM NaCl and 10 mM DTT in 10 mM Tris, pH 5.5. (**D**,**E**), Lanes 1 through 6, in 20 mM NaCl and 10 mM DTT in 10 mM Tris, pH 5.5; Lanes 7 through 11, in 20 mM NaCl and 10 mM DTT in 10 mM Tris, pH 7.4. (**D**), Lane M, protein molecular weight standards; Lanes 1 and 7, oHb alone; Lanes 2 through 6, and 8 through 12, oHb with mHcCPs which include mHcCBP1 (Lanes 2, 8), mHcCBP2 (Lanes 3, 9), mHcCP1a (Lanes 4, 10), mHcCP1b (Lanes 5, 11), and mHcCP1c (Lane 6, 12). (**E**), Lane M, protein molecular weight standards; Lanes 1 and 7, BSA alone; Lanes 2 through 6, and 8 through 12, BSA with mHcCPs which include mHcCBP1 (Lanes 2, 8), mHcCBP2 (Lanes 3, 9), mHcCP1a (Lanes 4, 10), mHcCP1b (Lanes 5, 11), and mHcCP1c (Lane 6, 12).

To investigate the effect of the reducing agent dithiothreitol (DTT) on the activity of cysteine proteases, a degradation assay was performed at pH 5.5 and pH 7.4 in the presence or absence of DTT. All mHcCPs remained active in degrading oFg in the presence of a reducing agent at pH 7.4 at 37 °C and 42 °C; however, except for mHcCBP2, their oFg-degrading activities diminished at pH 5.5 in the presence of a reducing agent (Figure 4A,B, Supplementary Figure S8). The presence of DTT either did not affect or slightly increased the activity of mHcCBP2 on oFg (Figure 4A,B). Additionally, at pH 5.5, all mHcCPs were found to degrade oHb partially (Figure 4D). Notably, mHcCBP2 was capable of completely degrading both oHb and BSA at pH 5.5 and pH 7.4 in the presence of DTT (Figure 4D,E) and partially breaking down bIgG at pH 5.5 in the presence of DTT (Figure 4C).

### 2.5. Time-, Dose-, and Temperature-Dependent Fibrinogenolytic Activity of mHcCPs

**Figure 5:**
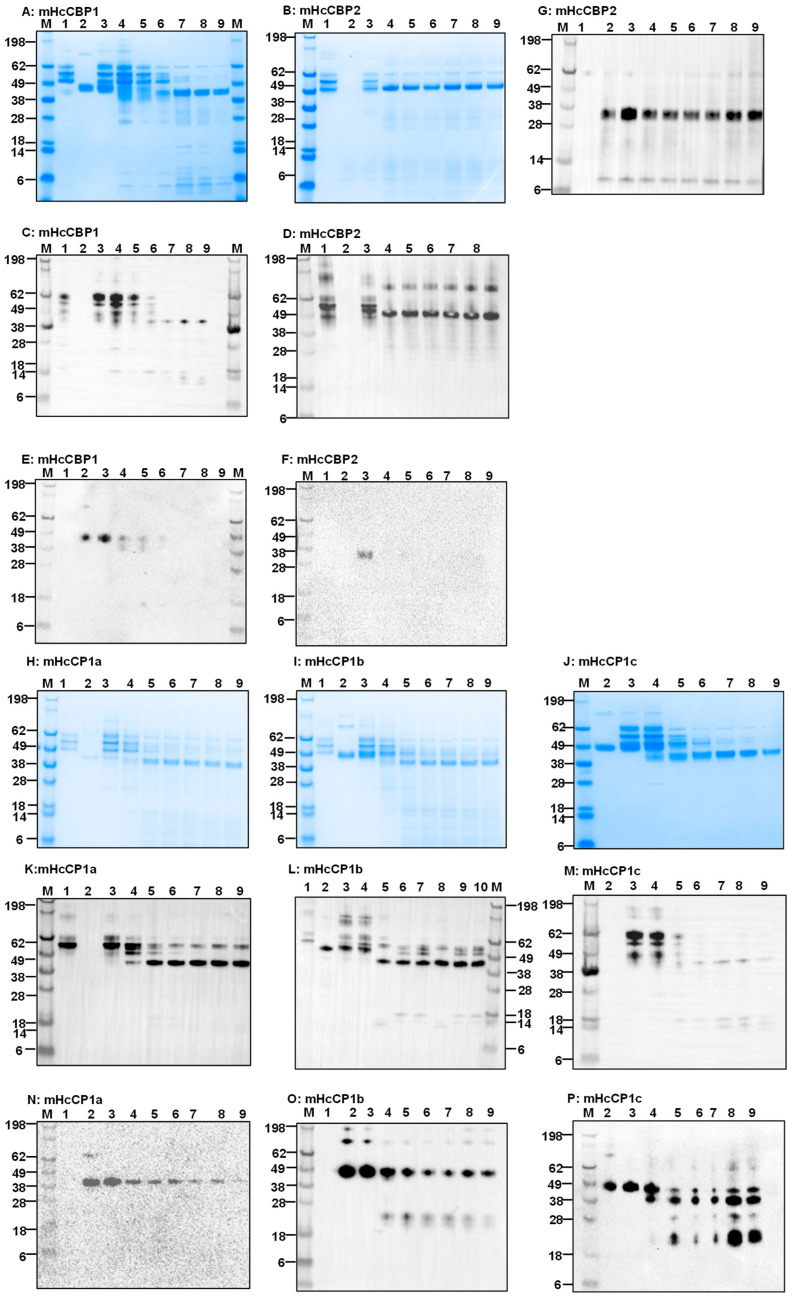
The analysis of time-dependent degradation of ovine fibrinogen (oFg) by mHcCPs. (**A**–**G**): SDS-PAGE (**A**,**B**) and Western blot (**C**–**G**) by mHcCBP1 (**A**,**C**,**E**) and mHcCBP2 (**B**,**D**,**F**,**G**) at 37 °C in PBS, pH 7.4. Lane 1, oFg alone; Lanes 2, mHcCBP1 alone (**A**,**C**,**E**) or HcCBP2 alone (**B**,**D**,**F**). Aliquots were collected from the digestion for analysis at 0 h (Lane 3), 3 h (Lane 4), 6 h (Lane 5), 9 h (Lane 6), 12 h (Lane 7), 15 h (Lane 8), and 18 h (Lane 9) in incubation. (**A**,**B**), SDS-PAGE with Coomassie blue staining; (**C**,**D**), Western blotting using the anti-bovine Fg antibody; (**E**,**F**), Western blotting using an anti-His antibody for detection of mHcCBP1 and mHcCBP2, respectively; (**G**), Western blotting using an anti-HcCBP2 antibody for detection of mHcCBP2. SDS-PAGE was performed under reducing conditions. (**H**–**P**): SDS-PAGE (**H**,**I**,**J**) and Western blot (**K**–**P**) analysis of time-dependent degradation of oFg by mHcCP1a (**H**,**K**,**N**), mHcCP1b (**I**,**L**,**O**) and mHcCP1c (**J**,**M**,**P**) at 37 °C in PBS, pH 7.4. Lane 1, oFg alone; Lanes 2, mHcCP1a (**H**,**K**,**N**) or mHcCP1b (**I**,**L**,**O**) alone or mHcCP1c (**J**,**M**,**P**) alone. Aliquots were collected from the digestion for analysis at 0 h (Lane 3), 3 h (Lane 4), 6 h (Lane 5), 9 h (Lane 6),12 h (Lane 7), 15 h (Lane 8), and 18 h (Lane 9). (**H**–**J**), SDS-PAGE with Coomassie blue staining; (**K**–**M**), Western blotting using the anti-bovine Fg antibody; (**N**–**P**), Western blotting using an anti-His antibody for detection of mHcCP1a, mHcCP1b, and mHcCP1c, respectively.

To determine the temporal kinetics of Fg degrading activity of mHcCPs, oFg was co-incubated with mHcCBP1 (Figure 5A,C,E), mHcCBP2 (Figure 5B,D,F,G), mHcCP1a (Figure 5H,K,N), mHcCP1b (Figure 5I,L,O) and mHcCP1c (Figure 5J,M,P) in PBS at pH 7.4 and aliquots of the reaction mixture were collected at 0, 3, 6, 9, 12, 15, and 18 h for analysis. Degradation initiated with the cleavage of the mHcCPs themselves as early as 3 h of incubation (Figure 5A,B,H,I,J, Lane 4). By 6 h in incubation, significant degradation of oFg was observed (Figure 5A,B,H,I,J, Lane 5). As the incubation time increased, the mHcCP-mediated degradation of oFg increased. Notably, the oFg was reduced in molecular weight by mHcCPs within 12 to 15 h to below that of oFg gamma chain (Figure 5A,C for mHcCBP1, H & K for mHcCP1a, I & L for mHcCP1b, and J & M for mHcCP1c) except for mHcCBP2 which degraded oFg to a molecular weight equivalent to that of the gamma chain of oFg (Figure 5B,D). The C-terminus (His-tagged) of mHcCBP1 was depleted as early as 3 h during co-incubation with oFg; however, it remained intact when incubated alone throughout the incubation period (Figure 5E). In contrast, mHcCBP2 lost its C-terminus when cultured alone for 18 h or co-incubated with oFg for 3 h or longer (Figure 5F). The presence of mHcCBP2 (Figure 5G) without the C-terminus (His-tagged) was confirmed by antisera against HcCBP2. For mHcCP1a, b, and c, co-incubation with oFg caused a gradual loss of the C-termini over time. Yet, lower molecular weight fragments detected by the anti-His antibody were also apparent for mHcCP1b and c (Figure 5N,O,P). To demonstrate the synergistic effect of HcCPs, we compared the fibrinogenolytic activities of mHcCBP1 and mHcCP1c alone and in combination (Supplementary Figure S9). The results showed that combined mHcCBP1 and mHcCP1c (Supplementary Figure S9A, Lane 2) were significantly more potent in degrading oFg than any of the proteins alone (Supplementary Figure S9A, Lane 8 and 9; Supplementary Figure S9B). The dose-dependent degradation of oFg by mHcCBP2 showed that mHcCBP2, at a dose as low as 2 ng/µL, was able to degrade 66.6 ng/µL of oFg in PBS, pH 7.4, at 37 °C and 42 °C (Supplementary Figure S10A,B). The temperature-dependent degradation showed that mHcCBP1 was able to degrade oFg at temperatures ranging from 37.7 °C to 44.7 °C (Supplementary Figure S11A, Lanes 3–6) but not at 46.8 °C or 48.7 °C (Supplementary Figure S11A, Lanes 1 and 2). Yet, mHcCBP1 failed to degrade oHb at any temperature ranging from 37.7 °C to 48.7 °C (Supplementary Figure S11A, Lanes 10–15). Additionally both mHcCBP1 and mHcCP1c remained intact following 24 h incubation in PBS at 37 °C (Supplementary Figure S12, Lanes 1 and 6) and active in degrading oFg after incubation at 37 °C for 12 h with or without prior exposure to oFg (Supplementary Figure S12, Lanes 3, 5, 7, and 9).

### 2.6. mHcCBP1/mHcCP1c and oFg Native Structure-Based Interactions Are Required for Protease Activity

**Figure 6:**
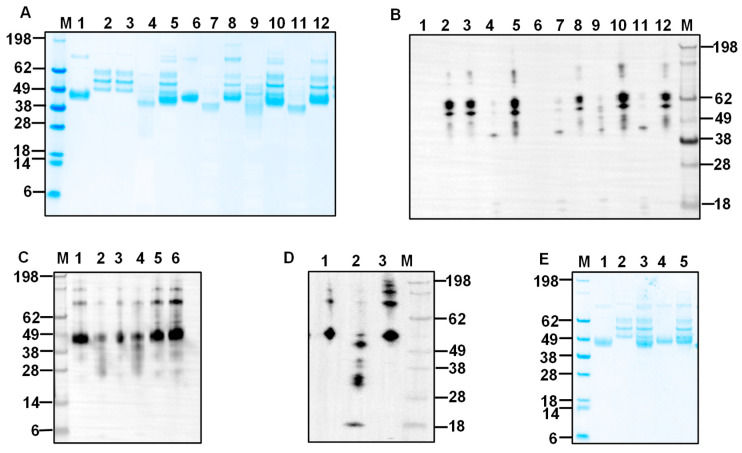
SDS-PAGE (**A**,**E**) and Western blot ((**B**), anti-bFg antibodies; (**C**), anti-HcCBP1 antibodies; and (**D**), anti-His antibodies) analysis of substrate degradation by mHcCBP1 and mHcCP1c under heat denaturation conditions at 80 °C for 5 min. (**A**), SDS-PAGE with Coomassie blue staining. Lane 1, intact mHcCBP1; Lane 6, intact mHcCP1c; Lane 2, intact ovine fibrinogen (oFg) alone; Lane 3, heat-inactivated oFg alone; Lanes 4 and 9, intact mHcCBP1 and intact oFg; Lanes 5 and 10, intact mHcCBP1 and heat-inactivated oFg; Lanes 7 and 11, intact mHcCP1c and intact oFg; Lanes 8 and 12, intact mHcCP1c and heat-inactivated oFg. Panels (**A**,**B**) have the same lane layout. (**C**), Lane 1 intact mHcCBP1 alone; Lanes 2 and 4, intact mHcCBP1 and intact oFg; Lanes 3 and 5, intact mHcCBP1 and heat-inactivated oFg. Lane 6, heat-inactivated mHcCBP1 alone. (**D**), Lane 1, intact mHcCP1c alone; Lane 2, intact mHcCP1c and intact oFg; Lane 3, intact HcCP1c and heat-inactivated oFg. (**E**), SDS-PAGE with Coomassie blue staining. Lane 1, heat-inactivated mHcCBP1; Lane 2, intact oFg alone; Lane 3, heat-inactivated mHcCBP1 and intact oFg; Lane 4, heat-inactivated mHcCP1c; Lane 5, heat-inactivated mHcCP1c and intact oFg.

To assess whether protein substrate structural integrity is required for CP activities, we performed experiments determining the capability of HcCPs to degrade their substrate when enzymes and/or substrates were heat-denatured. Heat inactivation of enzyme or substrate abolished degradation of oFg (Figure 6A–E). Heat deactivation abolished mHcCP activities (Figure 6E).

### 2.7. Inhibition of the Fibrinogenolytic Action of mHcCPs by E-64 Protease Inhibitor

**Figure 7:**
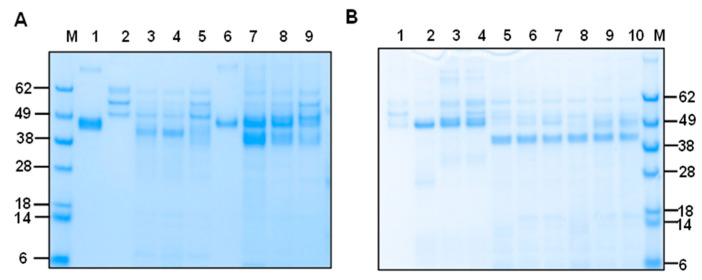
E-64 inhibition of the fibrinogenolytic action of mHcCPs. Proteins were analyzed by SDS-PAGE followed by Coomassie blue staining. mHcCPs were pre-incubated with two different concentrations of E-64 for 15 min at 37 °C prior to the addition of ovine fibrinogen (oFg), the mixtures were further incubated for 16 h at 37 °C at pH 7.4. (**A**). Lane M, protein standards; Lane 1, intact mHcCBP1; Lane 2, oFg; Lane 3, oFg and mHcCBP1; Lane 4, oFg and mHcCBP1 in the presence of 10 µM of E-64; Lane 5, oFg and mHcCBP1 in the presence of 50 µM of E-64; Lane 6, mHcCP1c alone; Lane 7, oFg and mHcCP1c; Lane 8 oFg and mHcCP1c in the presence of 10 µM of E-64; Lane 9, oFg and mHcCP1c in the presence of 50 µM of E-64. (**B**). Lane M, protein standards; Lane 1, oFg; Lane 2, oFg and mHcCBP2; Lane 3, oFg and mHcCBP2 in the presence of 10 µM of E-64; Lane 4, oFg and mHcCBP2 in the presence of 50 µM of E-64; Lane 5, oFg and mHcCP1a; Lane 6, oFg and mHcCP1a in the presence of 10 µM of E-64; Lane 7, oFg and mHcCP1a in the presence of 50 µM of E-64; Lane 8, oFg and mHcCP1b; Lane 9, oFg and mHcCP1b in the presence of 10 µM of E-64; Lane 10, oFg and mHcCP1b in the presence of 50 µM of E-64.

To investigate whether the fibrinogenolytic activity of mHcCPs is inhibited by cysteine protease inhibitors, E-64, an irreversible cysteine protease inhibitor, was used in the degradation assay in PBS at pH 7.4 for 16 h at 37 °C. The results showed that there was a dose-dependent, mild to complete ablation of the fibrinogenolytic activities of mHcCBP1, mHcCP1c (Figure 7A) and mHcCBP2, mHcCP1a and mHcCP1b (Figure 7B).

### 2.8. The Effect of mHcCPs on Recalcification Time (RCT) of Human Reference Plasma

**Figure 8:**
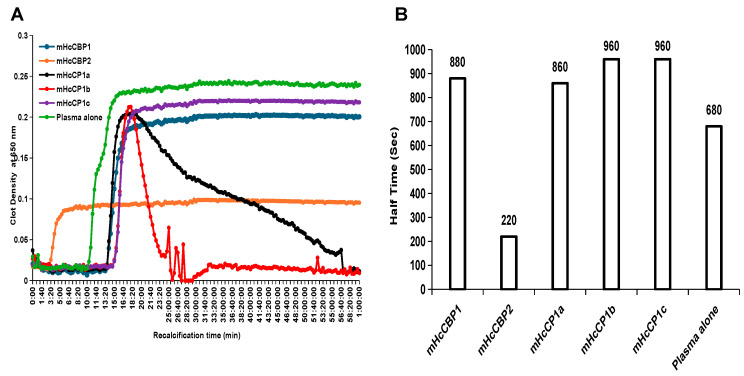
The effect of recombinant *H. contortus* cysteine proteases (mHcCPs) on plasma clotting as determined by recalcification time (**A**) and half-time (s) (**B**). The plates were read at 20 s intervals over a 60 min period using a spectrophotometer set at 37 °C. All assays were repeated 3 times in duplicate. Half-time is presented as the plasma clotting time needed to reach a half-maximum absorbance value.

In comparison to the RCT of human reference plasma alone, which had a clot initiation time of 10 min and 20 s, clot initiation was delayed by mHcCP1b or mHcCP1c for 4 min and 40 s, by mHcCP1a for 3 min 40 s, and by mHcCBP1 for 3 min and 20 s (Figure 8A). In contrast, mHcCBP2 promoted clot formation by initiating clotting 7 min earlier than the RCT of human reference plasma alone (Figure 8A).

mHcCP treatments resulted in varying levels of clot density in human reference plasma; therefore, we used the half-time to describe the time required to reach half-maximum absorbance, which is equivalent to the maximum clot density (Figure 8B). Pre-incubation with each mHcCP had a similar half-time, except for mHcCBP2, which had a half-time of 3 min and 40 s, accounting for a shorter clot initiation and lower maximum clot density. After incubation, the clot density of the plasma pre-incubated with mHcCPs was lower than that of the plasma alone (Figure 8A). While the clot density of plasma pre-incubated with mHcCBP1, mHcCBP2, or mHcCP1c remained constant, mHcCP1a and mHcCP1b lysed the plasma clot by a lysis time of 19 min for mHcCP1a and 3 min for mHcCP1b, respectively (Figure 8A).

### 2.9. Cysteine Protease Activity Using the Fluorogenic Peptide Substrate

**Figure 9:**
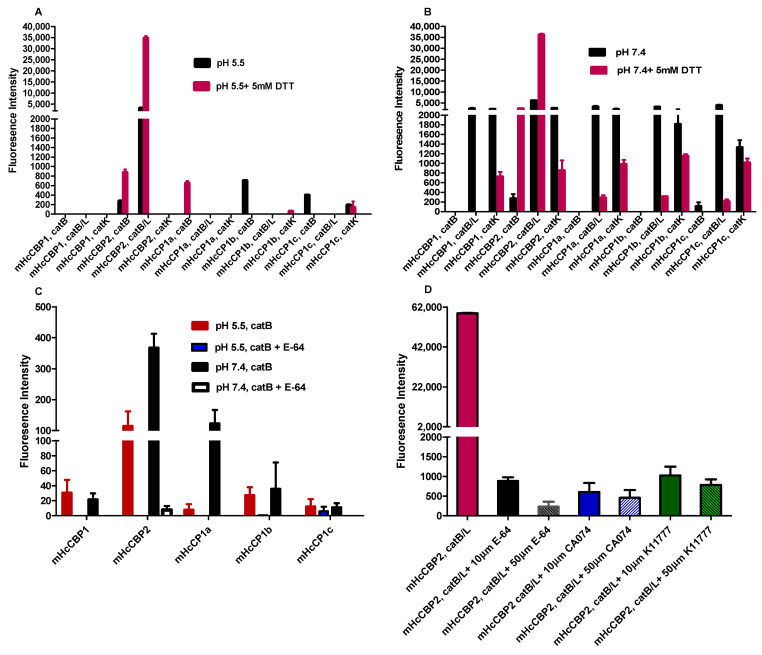
Cathepsin activity of recombinant *H. contortus* cysteine proteases (mHcCPs) was assayed using synthetic substrates including Z-Arg-Arg-AMC (catB), Z-Phe-Arg-AMC (catB/L), and Z-Leu-Arg-AMC (catK) at 37 °C for 60 min at pH 5.5 (**A**) and 7.4 (**B**) with or without 5 mM DTT. The inhibitory effect of the cysteine protease inhibitor, E-64, on all HcCPs with catB substrate at pH 5.5 and 7.4 (**C**) and E-64, catB (CA074) and catL (K11777) inhibitors on HcCBP2 with catB/L substrate was determined at pH 7.4 with 5 mM DTT (**D**). The fluorescence intensity in triplicate is expressed as mean ± SE.

All mHcCPs were tested for activities using substrates specific to catB (Z-arg-arg-AMC), catK (Z-Leu-arg-AMC) and catB/L (Z-phe-arg-AMC) [15]. mHcCBP2 was shown to be predominantly active in the presence of DTT at both pH 5.5 and 7.4 on the substrate specific to catB/L; however, it was also active on catK and catB with low activities at pH 7.4 (Figure 9B). The rest of the mHcCPs had low or no activities on any of the substrates tested (Figure 9A,B). In addition, we determined the effect of E-64 (cysteine protease inhibitor) on all mHcCPs using the catB substrate at pH 5.5 and 7.4 and showed that the ability of mHcCPs to enzymatically hydrolyze the catB substrate was inhibited by E-64 under both pH conditions (Figure 9C). Furthermore, we tested the effect of E-64 and catB and catL specific inhibitors on mHcCBP2 with catB/L substrate and demonstrated that all 3 inhibitors inhibited the activity of mHcCBP2 (Figure 9D).

### 2.10. Detection of Anti-HcCP Antibodies in Sheep Naturally Infected with H. contortus

**Figure 10:**
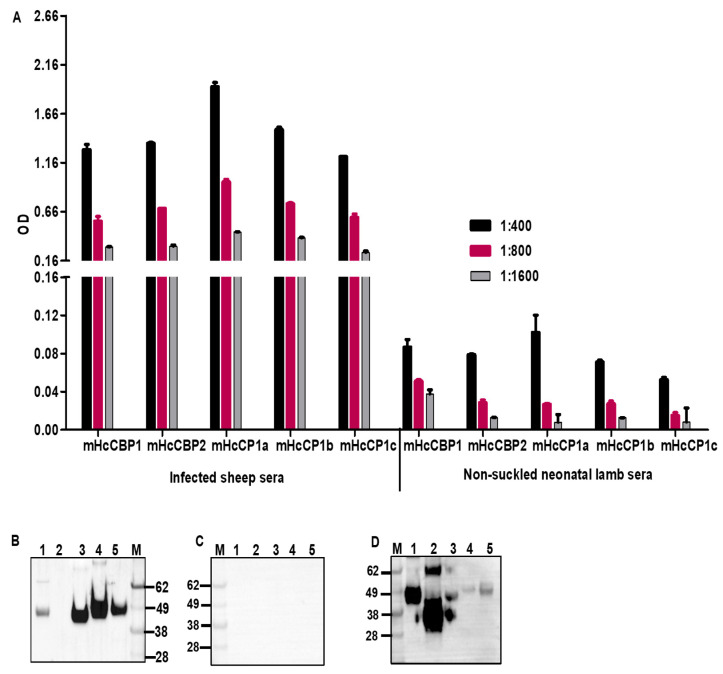
Detection of mHcCPs by ELISA (**A**) and Western blotting using pooled sera from sheep naturally infected with GI nematodes (**B**) or non-suckled neonatal lambs (**C**). Rabbit anti-HcCBP2 sera were used to detect mHcCBP2 in Western blotting (**D**). For Western blotting, Lane 1, mHcCBP1; Lane 2, mHcCBP2; Lane 3, mHcCP1a; Lane 4, mHcCP1b; Lane 5, mHcCP1c; Lane M, protein molecular standards.

To determine if sheep naturally infected with *H. contortus* responded to HcCPs immunologically, ELISA and Western blotting were employed to detect serum antibodies using mHcCPs. Sera pooled from 9 sheep naturally infected with *H. contortus* detected all mammalian cell-expressed HcCPs using ELISA (Figure 10A) but failed to detect HcCBP2 by Western blotting (Figure 10B). Pooled sera from 2 non-suckled neonatal lambs did not detect any of the HcCPs by either ELISA or Western blotting (Figure 10A,C). To confirm the presence or the integrity of mHcCBP2, which was not detected by the pooled sheep sera, rabbit anti-HcCBP2 sera were used to re-probe the same blot. We showed that mHcCBP2 was indeed detectable by the rabbit antisera (Figure 10D).

## 3. Discussion

The development of commercial vaccines for parasitic nematodes presents a substantial challenge due to the difficulty of expressing protective antigens in a form that can induce protective immunity [16]. This predicament is further compounded by the limited quantities of native proteins from the parasites, which restricts the possibility of vaccination studies. The availability of antigens in recombinant form is critical, as they can induce immunity while circumventing the need for native proteins. There is no commercially available recombinant vaccine against the highly pathogenic nematode parasites, such as the hematophagous *H. contortus*. It is, therefore, essential to identify the vaccine targets and explore the use of recombinant vaccine candidates in animal trials.

Nematode parasites typically exhibit a diminished metabolic complexity and are reliant on the host for the acquisition of nutrients. This dependency can result in considerable negative impacts on the host [17]. *H. contortus* produce cysteine proteases (HcCPs) promoting proteolytic breakdown of host proteins (including hemoglobin, immunoglobulins, fibrinogen, and extracellular matrix components), facilitating nutrient acquisition and immune evasion [18,19,20,21]. Given their importance during the parasitic stage, HcCPs have become a subject of intense research interest, where these proteases represent a promising avenue for further exploration in the field of host–parasite interactions [18,19,20]. In various vaccine trials, these proteases have been evaluated for their protective properties. Remarkably, the cysteine proteases from the adult *H. contortus* induce significant levels of protective immunity against homologous challenge in immunized lambs [20,21,22], warranting further exploration of the potential protective benefits of cysteine proteases [21]. Moreover, cysteine proteases, when used as vaccine candidates, confer protection against other blood-feeding gastrointestinal nematodes, such as hookworms (*Ancylostoma* spp. and *Necator americanus*) [13,23]. Although host homologues exist, parasite cysteine proteases have distinct structural and biochemical properties including, pH optima and stability, the alteration in peptide loops or domain extensions, diverse substrate specificity and cellular location. The disparate nature of parasite cysteine protease compared to the host orthologous proteins opens up opportunities for chemotherapy and/or vaccination.

Here, we demonstrate that HcCPs are produced by both L3 and adult *H. contortus*, suggesting that these cysteine proteases play a role during the life cycle. Most importantly, we showed that pooled sera from sheep naturally exposed to *H. contortus* had antibodies to HcCPs. The data indicate that these proteins are antigenic to the host and are secreted (given their signal peptide) during infection. It is intriguing that mHcCBP2 was not detected by Western blotting but by ELISA using the sera from sheep infected with *H. contortus*. This may be explained by the conformational epitopes being destroyed by denaturing and reduction in Western blot analysis. Alternatively, HcCBP2 was shown in this study to be unstable even when alone; its degradation is likely due to its ability to self-cleave [24]. Given that HcCBP2 is catL-like and highly active in degrading multiple host blood proteins, such self-cleavage is not only essential for its autoactivation but also for “self-clearance”, and may be a potential mechanism for parasite evasion from host surveillance.

In the present study, using the mammalian expression platform, we further characterized five different HcCPs, including HcCBP1 and HcCBP2, identified by Bakshi et al. (2021) [12], as well as HcCP1a, HcCP1b, and HcCP1c, which are *Haemonchus* orthologs to the hookworm vaccine antigen *Ancylostoma ceylanicum* CP1 [13]. As each of the 5 proteins contain a signal peptide, they presumably are secreted. Our data showed that there were differential expressions of HcCPs during distinct life cycle stages, e.g., HcCBP2, HcCP1a, b, and c in infective L3, whereas HcCBP1, HcCBP2, and HcCP1a were expressed in adult worms, indicating that these proteins may be crucial for the development of *H. contortus* throughout the parasitic phase. The variations in molecular weight reflect stage-specific proteolytic processing of the proteins. Cysteine proteases are initially synthesized in an inactive form referred to as zymogens, which contain a propeptide that is subsequently removed during the proteolytic process [25]. The native zymogen exhibits a higher molecular weight than the mature, active enzyme. Furthermore, proteins harboring signal peptides prior to enzymatic cleavage and secretion possess higher molecular weights in comparison to those of the secreted proteins [26]. In some cases, the protein may also be associated with an endogenous inhibitor, forming a complex [27]. Moreover, specific types of post-translational modifications can account for some of the heterogeneity in protein sizes [28]. The increased molecular weight observed at the L3 stage suggests the presence of either the full-length zymogen, a signal peptide, or a complex involving an inhibitor. Conversely, mature and active low molecular weight proteases in the worms at adult stage may reflect their intensified metabolic and reproductive demand.

In this study, the HcCPs were initially expressed in a bacterial expression system. Undesirably, none of the bHcCPs demonstrated activities in degrading the host blood protein substrates. The improper folding of the bacterial recombinant proteins and post-translational modifications may have contributed to the lack of activity [12], which may prevent self-cleavage into their active forms in the presence of a substrate. Similar findings have been reported in studies involving catL-like proteases from *Taenia solium* and *Gnathostoma spinigerum* [15,29], where the proteins expressed as recombinant proteins in *E. coli* failed to exhibit enzymatic activity. In contrast, these proteins, when expressed in eukaryotic systems, demonstrated the typical enzymatic activities associated with cysteine proteases. The superiority of mammalian cells for synthesizing biochemically active proteins from parasitic nematodes suggests that this system should be preferred for future efforts to synthesize antigens for vaccination against these parasites.

**Figure 11:**
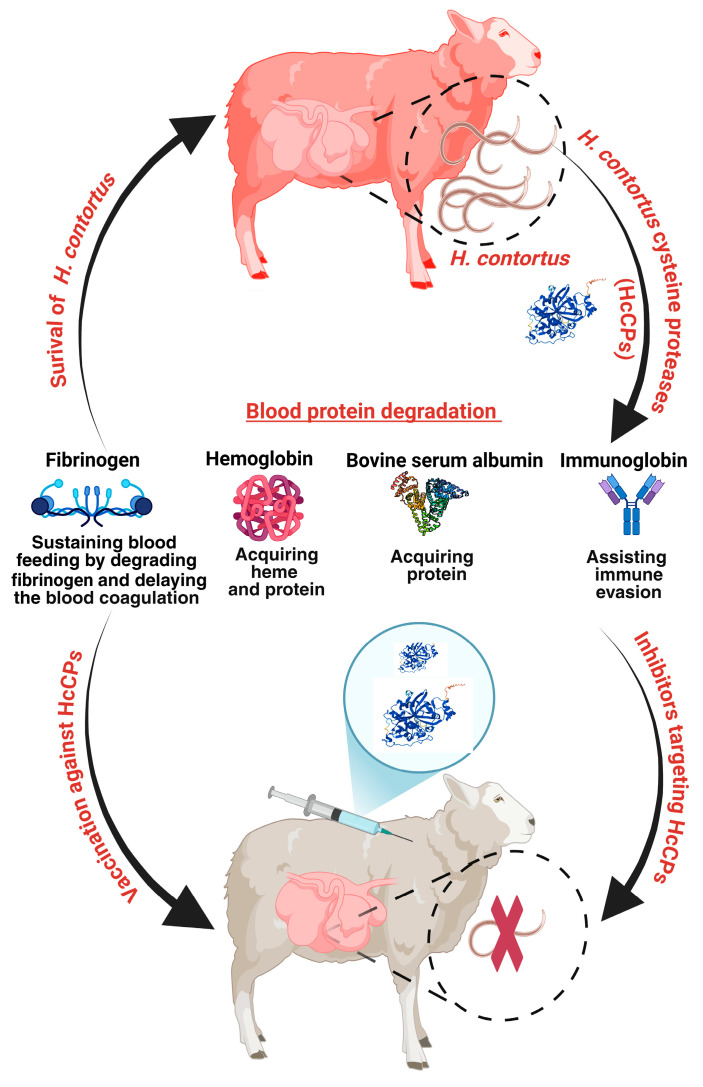
Schematic representation of *H. contortus* cysteine proteases (HcCPs) facilitating parasite survival in the host abomasum by sustaining blood feeding through modulation of host blood clotting and degradation of abundant plasma proteins for nutritional acquisition (Created with BioRender.com).

All mHcCPs degraded bFg, oFg, or pFg; they appeared most potent in degrading oFg. Species specific degradation of permissive host blood protein substrates by parasite cysteine proteases have been reported previously. These patterns are consistent with the hypothesis that blood-feeding parasitic nematodes have evolved digestive enzymes that are specifically optimized to digest proteins from their mammalian host species, rather than orthologous proteins from other non-host mammals [30,31,32,33]. Furthermore, mHcCBP2, but not mHcCBP1, mHcCP1a, mHcCP1b, or mHcCP1c, degraded oHb and BSA at a host physiological pH, indicating substrate specificity and selectivity. The results suggest that all HcCPs, except for HcCBP2, play a specific role in regulating blood clotting. At the same time, HcCBP2, with a broader substrate specificity, shows that it may play a potential role in the nutritional strategy of the parasite, by utilizing various host proteins for growth and survival (Figure 11) [34]. The mHcCPs exhibited activities at elevated temperatures, ranging from 42 °C to 44.7 °C, as well as remarkable stability when exposed to extended incubation at 37 °C, demonstrating their considerable thermostability in the ruminant host, which has higher body temperatures (38.6 to 39.7 °C) [35].

Moreover, the proteases were found to be most active at pH 5.5 and 7.4. These findings align with the behavior of native cysteine proteases found in the excretory-secretory products of *H. contortus* [19]. For example, during blood feeding, the parasite-secreted cysteine proteases are exposed to near neutral pH of the host blood at the feeding site. In contrast, when the parasite ingests blood proteins, it is exposed to an acidic environment in the gut of the parasite [36]. The human catB is distinctive among cathepsins and cysteine proteases due to its dual exopeptidase and endopeptidase cleavage capabilities. At an acidic pH, it functions as a lysosomal exopeptidase, specifically a carboxy-dipeptidase, cleaving only dimers from the C-terminal end. As the pH increases (cytoplasm), the catB acquires the ability to act as an endopeptidase. The differing peptidase functions of catB are regulated by a 20-amino acid segment known as the occluding loop. This loop, held in place by salt bridges to histidine residues H110 and H111 at lower pH levels, obstructs access to the binding groove and prevents endopeptidase activity. At higher pH, this loop becomes exposed. Therefore, the two protonated histidine residues in the occluding loop play a crucial role in promoting catB exopeptidase activity. Notably, human catB loses its activity at pH values exceeding 7.2 [14,37,38]. Our results show HcCBP1, HcCBP2 and HcCP1c possess the two histidine residues within the potential occluding loop, which may be the reason why these proteases retain catalytic activities across a broad pH range. Thus, the infection is capable of altering the pH from acidic to near-neutral conditions without hindering the activities of the cysteine proteases. This adaptability is essential for the parasite’s survival within its host. Our data showed that reducing conditions caused variations in mHcCP activities, particularly when fluorescent synthetic cathepsin substrates were used, which is in part consistent with the characteristics of canonical cathepsins in a redox environment [39]. Additionally, HcCBP2 also partially degrades IgG, most likely by fragmenting the heavy chain, which is consistent with the previous findings that helminth ES cysteine proteases degrade host IgG, thereby playing an essential role in immune evasion [40,41].

When cysteine proteases interact with their substrates, they form temporary and unstable complexes [42]. This binding creates a transition state that allows the proteases to facilitate the cleavage of peptide bonds. This interaction relies heavily on the specific structural characteristics and environmental conditions surrounding both the protease and its substrate. These factors can significantly influence the efficiency and specificity of the enzymatic reaction [43,44,45]. In our results, the degradation of Fg by mHcCPs initiated with the cleavage of mHcCPs themselves, suggesting an enzyme-substrate interaction that may lead to the autoactivation of the HcCPs. Moreover, the native structures of substrates are crucial to the catalytic activity of mHcCPs because heat-inactivation of substrates (e.g., Fg) completely abolished the ability of HcCPs to degrade Fg. Additionally, inhibition by E-64 confirms that these proteases operate through a cysteine-dependent catalytic mechanism [46], as E-64 is a selective irreversible inhibitor of cysteine proteases [47]. The differential inhibition of HcCPs by E-64 suggests that HcCPs represent a family of functionally divergent cysteine proteases [48,49].

Our data indicates also that all HcCPs interfered with the coagulation process. This interference arises from the degradation of Fg resulting in the formation of clots with reduced density and a delay in clot initiation. An exception is with HcCBP2, which significantly accelerates clot initiation. Our data suggest that partial degradation of Fg by HcCPs may impair fibrin polymerization and cross-linking, resulting in the formation of weak, loose, or low-density clots [50]. These findings correspond with earlier results that demonstrated the fibrinogenolytic activity of ficin [51]. Interestingly, while HcCBP2 accelerates clot formation as a procoagulant, the density of the clot formed in its presence is considerably lower. Previous studies have shown that clots consisting of fibrin gamma chains exhibit thinner fibers, smaller pores, and decreased stiffness [52,53,54].

Additionally, HcCP1a and HcCP1b resulted in complete clot lysis within one hour of incubation. Fibrin serves as the primary component of blood clots, and the lysis of the clots suggests that certain enzymes possess fibrin lysis properties comparable to those of host plasmin. In our case, this mechanism may operate through either the stimulation of host plasmins or the direct action of the HcCP proteins themselves, an area that virtue further investigation. It is interesting to note that these findings bear similarities to the effects observed with catL-type enzymes, FhCL1 and FhCL2, derived from *Fasciola hepatica*, which cleave fibrinogen α-, β-, and γ-chains, as well as fibrin [55]. These effects highlight the potential of HcCPs to manipulate host blood clotting at the site of parasite infection and feeding, thereby favoring continuous blood uptake (Figure 11). Further, these proteins may have potential medical or veterinary applications targeting blood clotting disorders.

By cathepsin assay, we demonstrate that HcCBP2 preferentially cleaves the substrate Z-Phe-Arg-AMC (catB/L substrate), but does not readily hydrolyze Z-Arg-Arg-AMC, which is a preferred substrate of mammalian catB [56]. Thus, HcCBP2 is considered a catL-like protein. Despite the sequence comparison shows that HcCBP2 is more aligned to catB than catL of *A. caninum* and *H. sapiens*, HcCBP2 activity was inhibited by all inhibitors specific to catL or catB or cysteine proteases. This disparity may indicate that HcCBP2, as a predominant catL with low catB activity, retains a structural similarity to catB at the enzymatic center, as it is inhibited by catB specific inhibitor while exhibiting substrate specificity akin to that of catL [57]. A comprehensive structural investigation is necessary to compare and establish a definitive conclusion regarding classification of cathepsins of various species.

## 4. Materials and Methods

### 4.1. Identification and Functional Prediction of HcCP1a-c

We downloaded proteomes of *Ancylostoma ceylanicum* [58] and *Haemonchus contortus* [59] from the WormBase-ParaSite database [60]. We used OrthoFinder [61] to identify protein-coding genes of these two species that were orthologous to one another; we also used Phobius [62] to identify genes whose encoded proteins had predicted N-terminal signal sequences directing classical protein secretion. These analyses identified 53 *H. contortus* genes whose protein products were putatively secreted and that were orthologs of *Acey*-CP1, a secreted cysteine protease of *A. ceylanicum* that we previously demonstrated to have activity as a vaccine subunit [13]. We further subdivided these *H. contortus* genes by maximum-likelihood phylogenetic analysis [63,64] revealing 17 of the 53 to comprise a gene subfamily most closely related to *Acey-CP1*. We used Salmon [65] to cross-correlate these 17 *H. contortus* genes with published intestinal RNA-seq expression data for *H. contortus* [66]. Three of these genes, like *Acey-CP1*, proved to be highly expressed in the *H. contortus* intestine; these were HCON_00005870, HCON_00005860, and HCON_00005830, which we respectively called *HcCP1a*, *HcCP1b*, and *HcCP1c*.

### 4.2. Amino Acid Sequence Alignment and Phylogenetic Construction

Multiple sequence alignment was performed and the phylogenetic tree was constructed using Clustal Omega Version 1.2.2 (https://www.ebi.ac.uk/jdispatcher/msa/clustalo, (accessed on 18 June 2025) [67].

### 4.3. Recombinant H. contortus Cysteine Proteas e (rHcCP) Production

We identified two genes encoding the *H. contortus* cysteine proteases, cathepsin-B-like proteins 1 and 2 (HcCBP1, HcCBP2), with Genbank accession numbers CDJ83387.1 and CDJ87123.1, respectively [12], and three genes encoding the cysteine protease (CP) 1a, 1b, and 1c (HcCP1a, HcCP1b, and HcCP1c), with Genbank accession numbers CDJ88838.1 (99% amino acid identity), XGW24187.1, and CDJ88843.1, respectively. All five genes were cloned into pET29b+ (Novagen, Madison, WI, USA) for bacterial expression and pcDNA3.1 (Invitrogen, Waltham, MA, USA) for FreeStyle 293-F cells expression (Invitrogen, Waltham, MA, USA) with the addition of a His tag at the C-termini of all proteins for detection and purification purposes (Biomatik Corporation, Kitchener, ON, Canada). Moreover, the native signal peptide present in these HcCPs was replaced with the CD5 signal sequence to optimize secretion efficiency in FreeStyle 293-F cells.

For bacterial expression, the HcCBP1 and HcCBP2 constructs were transformed into the BL21 strain of *E. coli* (Agilent, Santa Clara, CA, USA), and expression was carried out in Luria-Bertani broth supplemented with 50 µg/mL of kanamycin and 3% ethanol [68]. Expression was induced by isopropyl-β-D-thiogalactopyranoside. The bacterial lysate was prepared for protein purification. The purified bHcCP1a, bHcCP1b, and bHcCP1c proteins were prepared by the Biomatik Corporation (Kitchener, ON, Canada). For production in the mammalian expression system, the recombinant plasmids were propagated in XL2-Blue ultra bacterial competent cells (Agilent, Santa Clara, CA, USA), purified using the Qiagen Maxi Prep kit (Qiagen, Germantown, MD, USA), and transfected into FreeStyle 293-F cells using PEI MAX (Polysciences, Warrington, PA, USA). Briefly, the mixture of plasmid DNA and PEI MAX was incubated for 15 min before being added to the cells, followed by incubation at 80% humidity, 8% CO_2_, and 37 °C on a shaker at 135 rpm. After 24 h of incubation, the culture volume was doubled by adding fresh medium, along with addition of 3.75 mM final concentrations of valproic acid. (MilliporeSigma, Burlington, MA, USA). The cells were then incubated for an additional 5 to 6 days. Aliquots of the culture supernatant were collected every 24 h to monitor cell viability and conduct protein expression analysis using SDS-PAGE and Western blotting using anti-His antibody. Upon reaching cell viability of 65–70%, the supernatant was harvested and centrifuged at 3500 RCF for 30 min at 4 °C before purification.

### 4.4. Purification of rHcCPs

Proteins were purified using Ni-NTA agarose (Qiagen, Germantown, MD, USA). For bacterial recombinant proteins, the cell lysate in binding buffer (50 mM NaH_2_PO_4_, pH 8; 300 mM NaCl, and 10 mM imidazole) was mixed with pre-equilibrated Ni-NTA agarose and incubated at 4 °C overnight on a rotater. Following incubation, the agarose beads were packed into a Poly-Prep chromatography column (Qiagen, Germantown, MD, USA). The column was washed with wash buffer 1 (50 mM NaH_2_PO_4_, pH 8; 300 mM NaCl, and 20 mM imidazole) and wash buffer 2 (50 mM NaH_2_PO_4_, pH 8; 300 mM NaCl, and 50 mM imidazole). The protein was eluted with 10 mL of elution buffer 1 (50 mM NaH_2_PO_4_, pH 8; 300 mM NaCl, and 250 mM imidazole) at 1 mL per fraction as per the manufacturer’s protocol. The purified protein fractions were pooled, dialyzed, and stored at −80 °C until use. Protein concentrations were determined by the NanoDrop 2000 spectrophotometer (Thermo Fisher Scientific, Waltham, MA, USA). The same purification procedure was used to purify mHcCPs from cell supernatants containing secreted proteins. Antisera against HcCBP1 and HcCBP2 were generated in rabbits [12] and antisera against HcCP1a, b, and c were generated in C57BL/6 mice using standard immunization protocols. Sera collected from sheep naturally infected with *H. contortus* were used as a source for anti-*H. contortus* antibodies against HcCPs. The animal use and care protocol was approved by the Institutional Animal Use and Care Committees (IACUC).

### 4.5. SDS-PAGE, Coomassie Blue Staining and Western Blotting

The protein in the sample buffer (Thermo Fisher Scientific, Waltham, MA, USA) was heated at 95 °C for 5 min and then separated using a 4–20% NuPAGE gel (Thermo Fisher Scientific, Waltham, MA, USA). Following electrophoresis, the gel was washed thrice with deionized water and stained with Simply-blue Safe Stain (Thermo Fisher Scientific, Waltham, MA, USA) for 1 h and destained with water for 1–3 h. To perform Western blotting, the proteins were transferred onto a polyvinylidene difluoride membrane (Mill poreSigma, Burlington, MA, USA), and the blot was subsequently blocked using 3% skim milk in PBS containing 0.1% Tween 20. The blot was then probed with a primary antibody followed by a horse radish peroxidase-conjugated secondary antibody and developed using the Super Signal West Dura Extended Duration horseradish peroxidase substrate (Thermo Scientific, Waltham, MA, USA), and the image was acquired using the ChemiDoc System (Bio-Rad, Hercules, CA, USA).

### 4.6. Preparation of Worm Extracts

Tissue extracts of stage 3 larvae (L3) and adult *H. contortus* were prepared from the frozen worms using a pulverizing mill (SPEX SamplePrep Freezer/Mills 6875, Metuchen, NJ, USA). The resulting powder was then dissolved in PBS and subjected to centrifugation at 13,300 RCF for 30 min. The supernatant, which contained soluble proteins, was collected and protein concentrations were quantified using the Bradford protein assay kit (Bio-Rad, Hercules, CA, USA). The remaining pellet (insoluble) and supernatant were kept at −80 °C until used.

### 4.7. Degradation of Blood Protein Substrates by rHcCPs—the Degradation Assay

The host blood proteins, namely ovine hemoglobin (oHb), bovine serum albumin (BSA), bovine immunoglobin G (bIgG), and bovine, ovine, and porcine fibrinogen (bFg, oFg, and pFg), were used as substrates to evaluate the proteolytic activity of the parasite cysteine proteases in our degradation assay as previously reported with modifications [18]. In brief, protein substrates (Millipore Sigma, Burlington, MA, USA) were separately dissolved at a concentration of 2–4 mg/mL in PBS (Thermo Fisher Scientific, Waltham, MA, USA) prior to use. Aliquots (1 µL) of each substrate were mixed with 1 µL containing 1–4 µg of HcCPs. The total reaction volume was adjusted to 30 µL and incubated at 37 °C for 16–18 h. The digestion reactions were stopped by adding SDS-PAGE sample buffer, and the protein digests were analyzed by SDS-PAGE with Coomassie blue staining.

### 4.8. pH-, Time-, Dose- or Temperature-Dependent Degradation of Protein Substrates by rHcCPs

The effect of pH on protein substrate hydrolysis by HcCPs was determined by using the following buffers: 0.1 M acetate (pH 4.0 and 5.5); 10 mM Tris with 20 mM NaCl (pH 5.5) with or without dithiothreitol (DTT); 0.1 M phosphate (pH 6.5); PBS (pH 7.4) with or without DTT; 0.02 M Tris (at pH 8.0); and cathepsin assay buffer (pH 7.0–8.0, Abcam, Cambridge, UK). In a total reaction volume of 30 µL, 1 µL (4 µg) of the substrate, 1 µL (1 µg) of HcCPs, and 28 µL of each of the buffers were included. The reaction was carried out for 16 h at 37 °C or 42 °C.

For time-dependent experiments, a total reaction volume of 50 µL comprising 10 µg of mHcCPs and 40 µg of oFg was incubated at 37 °C in PBS (pH 7.4) for 18 h. Aliquots (5 µL) were collected at 0, 3, 6, 9, 12, 15, and 18 h to monitor digestion. To evaluate the synergistic effect, both mHcCP1c and mHcCBP1 (5 µg each) were combined with 40 µg of oFg, and the same time course analysis was conducted.

Additionally, for dose-dependent degradation, different concentrations of mHcCBP2 ranging from 4 µg to 62.5 ng were incubated with a constant concentration of 2 µg of oFg at 37 °C or 42 °C for 16 h. For temperature-dependent degradation of oFg and oHb by mHcCBP1, the reaction was carried out at temperatures ranging from 37.7 °C to 48.7 °C. In addition to the pH-dependent experiments, all assays were carried out in PBS at pH 7.4. The reaction was terminated by adding a sample buffer containing a reducing agent and analyzed by SDS-PAGE, and proteins were visualized using Coomassie blue staining and/or Western blotting.

### 4.9. Native Structures as Requirements for mHcCPs to Degrade Fg

To examine if the native structures of both mHcCPs and Fg are required for Fg degradation, oFg was heat-denatured at 80 °C for 5 min prior to incubation with either native or heat-denatured mHcCBP1 or mHcCP1c. Controls included inactivation of mHcCBP1 and mHcCP1c. The reaction was performed either in PBS (pH 7.4) or cathepsin assay buffer (pH 7.0–8.0) for 16 h at 37 °C. The resulting degradation products were analyzed using SDS-PAGE with Coomassie blue staining and Western blot analysis.

### 4.10. Inhibition of Fibrinogenolytic Action of mHcCPs by a Cysteine Protease-Specific Inhibitor, E-64

E-64, L-trans-epoxysuccinyl-leucyl-amido (4-guanidino) butane (MilliporeSigma, Burlington, MA, USA) an irreversible mHcCP inhibitor that targets cysteine proteases, was used in this experiment [28]. mHcCPs were subjected to pre-incubation with E-64 at two concentrations (10 and 50 µM) for 15 min at 37 °C. Following pre-incubation, oFg was added to the mixture, followed by incubation at 37 °C for an additional 16 h in PBS, pH 7.4. The resulting protein degradation products were analyzed by SDS-PAGE and visualized by Coomassie blue staining.

### 4.11. Enzymatic Stability of mHcCBP1 and mHcCP1c

The mHcCBP1 or mHcCP1c protein alone, as well as with oFg, was incubated in PBS at pH 7.4 for 12 h. After incubation, oFg was added, and the incubation was continued for an additional 12 h. After the final incubation, the digests were analyzed by SDS-PAGE with Coomassie blue staining.

### 4.12. The Anticoagulant Function of mHcCPs

The effect of mHcCPs on blood clotting was evaluated using a modified recalcification time (RCT) assay, in which citrated plasma is supplemented with calcium chloride (CaCl_2_) to restore calcium levels, thereby activating the coagulation cascade. This activation can lead to the generation of thrombin, which in turn converts fibrinogen to fibrin, resulting in clot formation [69]. If fibrinogen structures are impacted, it may disrupt the kinetics of fibrin polymerization, which directly affects clotting time. Briefly, the RCT assay was performed by preincubating 50 μL of universal coagulation reference human plasma (UCRP) (DiaPharma, Louisville, KY, USA) with or without mHcCPs in 30 μL of 100 mM HEPES, pH 7.4, containing 150 mM sodium chloride at 37 °C for 15 min, followed by adding 20 μL of 50 mM CaCl_2_ to the reaction mixture. Incubation at 37 °C was continued for up to 1 h, and clotting was monitored at 650 nm at 20 s intervals using a spectrophotometer (SpecctraMax 190, Molecular Devices, San Jose, CA, USA). The time required to reach a half-maximum absorbance value, referred to as half-time [70], was used to describe the progressive plasma clotting over time. The clot initiation time was defined as the time from the addition of CaCl_2_ to the exponential rise in optical density above the baseline, which was the mean baseline absorbance value plus 1 standard deviation. Lysis time (LT) is the duration from the final turbidity, which corresponds to the maximum absorbance of the formed clot, to the time when 50% of the clot has lysed [71].

### 4.13. Cysteine Protease Activity Using Fluorogenic Peptide Substrates

The hydrolysis of the AMC (7-amino-4-methyl coumarin) fluorophore-containing substrates, Z-arg-arg-AMC (catB), Z-phe-arg-AMC (catB/L) (MilliporeSigma, Burlington, MA, USA) and Z-Leu-arg-AMC (catK) (Bachem Americas, Torrance, CA, USA) were conducted in a microtiter plate format using a fluorimeter (FluoStar Optima microplate reader, BMG Labtech, Ortenberg, Germany). In a standard assay, mHcCPs were prepared at a concentration of 0.25 µM in buffers with a pH of 5.5 (20 mM MES) or pH 7.4 (PBS), with or without DTT. The samples were incubated for 15 min at 37 °C before the reaction was initiated by adding the substrate to a final concentration of 50 µM. The total reaction volume was 200 µL. The reaction was monitored at an excitation and emission wavelengths of 355 and 460 nm at a 15 min interval for 1 h.

In the inhibition experiment, the inhibitors used are L-trans-epoxy succinyl-leucyl-amido(4-guanidino) butane (E-64) (MilliporeSigma, Burlington, MA, USA), an irreversible inhibitor that targets cysteine proteases, N-methyl piperazine-urea phenylalanyl homophenylalanyl-vinyl sulfone-benzene (K11777) (Adipogen Corporation, San Diego, CA, USA), an irreversible inhibitor specific to cathepsin L-like cysteine proteases; and N (L-trans-propylcaramoyloxirane-2-carbonyl)-L-isoleucyl-proline(CA074) (MilliporeSigma, Burlington, MA, USA) which inhibits catB-like cysteine proteases [15]. The mHcCPs and inhibitor were pre-incubated together in buffer in the absence of substrate for 5 min prior to the assay. All assays were carried out in triplicates.

### 4.14. Indirect Enzyme Linked Immunosorbent Assay (ELISA)

To detect antibodies against HcCPs, the recombinant proteins (mHcCBP1, mHcCBP2, mHcCP1a, mHcCP1b, and mHcCP1c) were diluted to 200 ng in 100 µL using an ELISA coating buffer (sodium bicarbonate buffer, pH 9.0), and then loaded onto 96-well plates for 1 h incubation at 37 °C followed by overnight incubation at 4 °C. The plates were blocked with a blocking buffer (PBS containing 0.05% Tween-20 and 5% horse serum). Afterward, the wells were incubated with test sera diluted at 1:400, 1:800 and 1: 1600 in 1× blocking buffer. All samples were assayed in duplicate. Following a wash step, the plates were incubated for 1 h with horseradish peroxidase (HRP) conjugated donkey anti-sheep secondary antibody at a dilution of 1:2000 (Thermo Fisher Scientific, Carlsbad, CA, USA). The wells were then washed again with PBS-Tween-20. 1-Step™ TMB ELISA Substrate Solutions (Thermo Fisher Scientific, Carlsbad, CA, USA) was added, and the reaction was incubated at room temperature for 25 min before being stopped with 2N sulfuric acid. The plates were read at 450 nm using a spectrophotometer (SpectraMax 190, Molecular Devices, San Jose, CA, USA).

## 5. Conclusions

In the present study, we identified *H. contortus* cysteine proteases exhibiting fibrinogenolytic (HcCBP1 and 2, HcCP1a, b, and c), anticoagulant (HcCBP1, HcCP1a, b, and c), procoagulant (HcCBP2), and profibrinolytic (HcCP1a and HcCP1b) properties [54]. These results indicate that HcCPs interfere with blood clotting before the clot is formed and advance degradation after the clot is formed. These proteins are produced by L3 and adult *H. contortus.* The sera from naturally infected sheep have antibodies specific to the 5 HcCPs, strongly suggesting that these proteases are released by the parasite and elicit host immune responses. Findings regarding the preservation of essential functional characteristics in mammalian recombinant HcCPs aligns with those of the native counterparts in the ES products of *H. contortus* [19]. These multi-functional proteases not only modulate blood clotting at different stages in the parasite’s favor but also broadly degrade other abundant plasma proteins for nutritional purposes. These findings highlight the critical role of cysteine proteases in parasitic survival and nutritional strategies, suggesting their potential as vaccine and therapeutic targets. This approach presents a promising alternative to chemical anthelmintics, addressing the growing issue of drug resistance in livestock gastrointestinal parasites.

## Data Availability

All data associated with this manuscript are available for access.

## Supplementary Materials

The following supporting information can be downloaded at: https://www.mdpi.com/article/10.3390/ijms262412077/s1.

## Abbreviations

bHcCPs	Bacterial recombinant *Haemonchus contortus* cysteine proteases
BSA	Bovine Serum Albumin
bFg, oFg, or pFg	Bovine, ovine, or porcine fibrinogen
DTT	Dithiothreitol
E-64	L-trans-epoxysuccinyl-leucyl-amido (4-guanidino) butane
Fg	Fibrinogen
GI	Gastrointestinal
Hc	*Haemonchus contortus*
HcCPs	*Haemonchus contortus* cysteine proteases
HcCBP1 and 2	*Haemonchus contortus* cathepsin B-like protein 1 and 2
HcCP1a, 1b, 1c	*Haemonchus contortus* cysteine protease 1a, 1b, 1c
Hb	Hemoglobin
HEPES	4- (2-hydroxyethyl)-1-piperazineethanesulfonic acid
IgG	Immunoglobulin G
IACUC	Institutional Animal Care and Use Committee
LT	Lysis Time
L3, L4, L5	Third, fourth, and fifth stage larvae
MES	2- (N-morpholino) ethanesulfonic acid
mHcCPs	Mammalian recombinant HcCPs
Ni-NTA	Nickel-Nitrilotriacetic Acid
OD	Optical Density
PBS	Phosphate Buffered Saline
RCT	Recalcification Time
RCF	Relative centrifugal force
rHcCP	Recombinant *H. contortus* cysteine protease
SDS-PAGE	Sodium Dodecyl Sulfate Polyacrylamide Gel Electrophoresis
Tris	Tris (hydroxymethyl) aminomethane
UCRP	Universal Coagulation Reference Plasma
