# Supplementary material for: Catalytically Active Recombinant Cysteine Proteases of Haemonchus contortus: Their Ability to Degrade Host Blood Proteins and Modulate Coagulation
Source: Int J Mol Sci. 2025 Dec 16;26(24):12077. doi: 10.3390/ijms262412077 (PMC12732675; doi:10.3390/ijms262412077)
Supplement: Supplementary file 1 [file ijms-26-12077-s001.zip › ijms-3934686-supplementary.pdf]

A

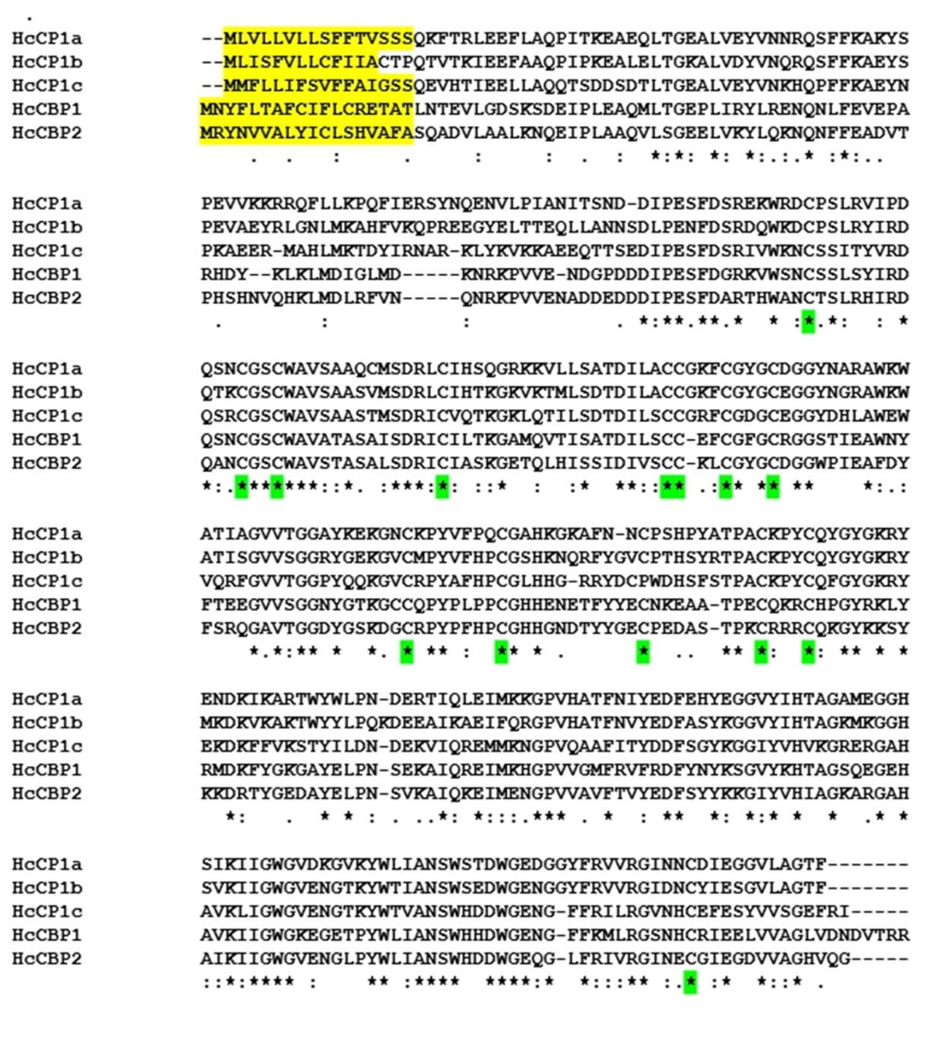

B

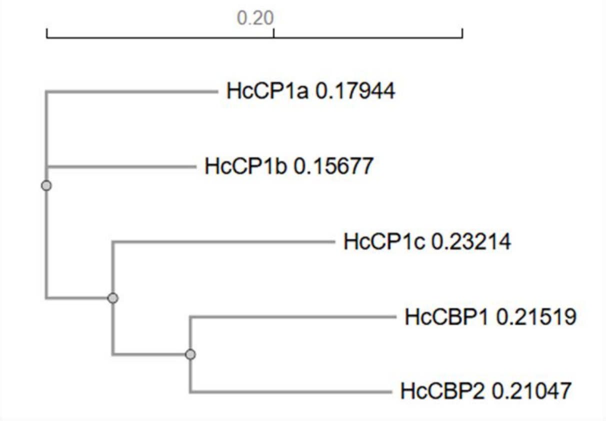

Supplementary Figure S1. Protein sequences were aligned (A) with Clustal Omega, and the phylogenetic tree (B) was generated from the resulting alignment. Conserved cysteine residues and signal peptide sequences are highlighted (A).

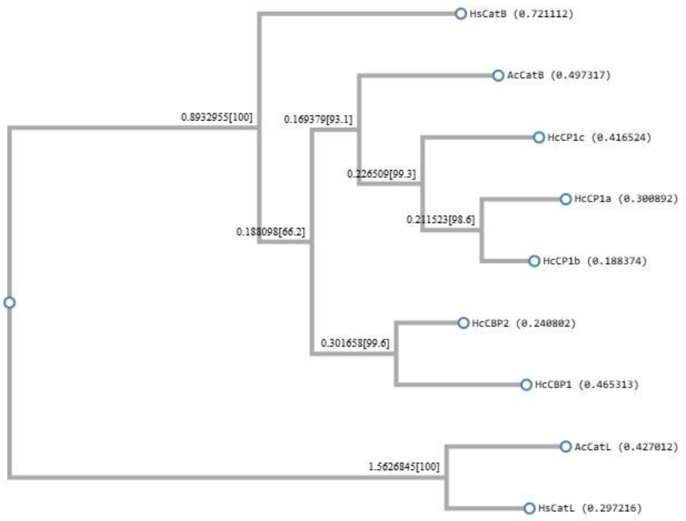

Supplementary Figure S2. Phylogenetic tree of cathepsin protein sequences. AccatB (RCN47340) and AccatL (AAL37181) are the cathepsins B and L from *A. caninum*, HscatB (AAH95408) and HscatL (KAI4007581) are the cathepsins B and L from *H. sapiens*.

CLUSTAL 2.1 multiple sequence alignment

```
HcCP1a          -------------MLVLLVLLSFFTVSSSQKFTRLEEFLAQPITKEAEQLTGEALVEYVN
HcCP1b          -------------MLISFVLLCFIIACTPQTVTKIEEFAAQPIPKEALELTGKALVDYVN
HcCP1c          -------------MMFLLIFSVFFAIGSSQEVHTIEELLAQQTSDDSDTLTGEALVEYVN
AcCatB          ----------------MWVFTALLVTTLAAKPTTVEEFLAQPVEEHVEQLTGQAFVDYIN
HcCBP1          -----------MNYFLTAFCIFLCRETATLNTEVLGDSKSDEIPLEAQMLTGEPLIRYLR
HcCBP2          -----------MRYNVVALYICLSHVAFASQADVLAALKNQEIPLAAQVLSGEELVKYLQ
HsCatB          ------------MWQLWASLCCLLVLANARSRPSFHPLS-------------DELVNYVN
HsCatL          MNPTLILAAFCLGIASATLTFDHSLEAQWTKWKAMHNRLYGMNEEGWRRAVWEKNMKMIE
AcCatL          ------------------------------------------------------------


HcCP1a          NRQSFFKAKYSPEVVKKRRQFLLKPQFIERSYNQENVLPIANITSND-DIPESFDSREKW
HcCP1b          QRQSFFKAEYSPEVAEYRLGNLMKAHFVKQPREEGYELTTEQLLANNSDLPENFDSRDQW
HcCP1c          KHQPFFKAEYNPKAEER-MAHLMKTDYIRNAR-KLYKVKKAEEQTTSEDIPESFDSRIVW
AcCatB          EHQSFYRAKYSPEAEAFVASRVMDSKFRRKPR----KEEVLSHVVRDEKLPESFDARQQW
HcCBP1          ENQNLFEVEPARHDY--KLKLMDIGLMDKNRK-----PVVE-NDGPDDDIPESFDGRKVW
HcCBP2          KNQNFFEADVTPHSHNVQHKLMDLRFVNQNRK-----PVVENADDEDDDIPESFDARTHW
HsCatB          KRNTTWQAGHNFYNVDMSYLKRLCGTFLGGPK-----PPQRVMFTEDLKLPASFDAREQW
HsCatL          LHNQEYREGKHSFTMAMNAFGDMTSEEFRQVMNGFQNRKPRKGKVFQEPLFYEAPRSVDW
AcCatL          ---------------------------------------------------------VDW
                                                                           *

HcCP1a          RDCPSLRVIPDQSNCGSCWAVSAAQCMSDRLCIHSQGRKKVLLSATDILACCGKFCGYGC
HcCP1b          KDCPSLRYIRDQTKCGSCWAVSAASVMSDRLCIHTKGKVKTMLSDTDILACCGKFCGYGC
HcCP1c          KNCSSITYVRDQSRCGSCWAVSAASTMSDRICVQTKGKLQTILSDTDILSCCGRFCGDGC
AcCatB          PKCKSIAIVRDQSACGSCWAVSSASAMSDELCVQSGGAINVVISDTDILSCCGEACGEGC
HcCBP1          SNCSSLSYIRDQSNCGSCWAVATASAISDRICILTKGAMQVTISATDILSCC-EFCGFGC
HcCBP2          ANCTSLRHIRDQANCGSCWAVSTASALSDRICIASKGETQLHISSIDIVSCC-KLCGYGC
HsCatB          PQCPTIKEIRDQGSCGSCWAFGAVEAISDRICIHTNAHVSVEVSAEDLLTCCGSMCGDGC
HsCatL          REKGYVTPVKNQGQCGSCWAFSATGALEGQMFRKTG--RLISLSEQNLVDCSGPQGNEGC
AcCatL          RDKGLVTEVKNQGMCGSCWAFSATGALEGQHARASG--QMVSLSEQNLVDCSTKYGNHGC
                 .   :  : :*  ******..:.  :...    :       :*  ::: *.    . **

HcCP1a          DGGYNARAWKWATIAGVVTGGAYKEKGNCKPYVFPQCGAHKGKAFN-NCPSHPYATPACK
HcCP1b          EGGYNGRAWKWATISGVVSGGRYGEKGVCMPYVFHPCGSHKNQRFYGVCPTHSYRTPACK
HcCP1c          EGGYDHLAWEWVQRFGVVTGGPYQQKGVCRPYAFHPCGLHHG-RRYDCPWDHSFSTPACK
AcCatB          EGGWPIEAYRWMKRDGVVTGGKYEEKNTCKPYAFYPCGKHKNAPYYGPCPRGSWPTPKCR
HcCBP1          RGGSTIEAWNYFTEEGVVSGGNYGTKGCCQPYPLPPCGHHENETFYYECNKEAA-TPECQ
HcCBP2          DGGWPIEAFDYFSRQGAVTGGDYGSKDGCRPYPFHPCGHHGNDTYYGECPEDAS-TPKCR
HsCatB          NGGYPAEAWNFWTRKGLVSGGLYESHVGCRPYSIPPCEHHVNGSRP--PCTGEGDTPKCS
HsCatL          NGGLMDYAFQYVQDNGGLDS------------------------------EESYPYEATE
AcCatL          NGGLMDLAFEYIKDNHGIDT------------------------------EESYPYVGRD
                 **    *: :      :

HcCP1a          PYCQYGYGKRYENDKIKARTWYWLPN-DERTIQLEIMKKGPVHATFNI-YEDFEHYEGGV
HcCP1b          PYCQYGYGKRYMKDKVKAKTWYYLPQKDEEAIKAEIFQRGPVHATFNV-YEDFASYKGGV
HcCP1c          PYCQFGYGKRYEKDKFFVKSTYILDN-DEKVIQREMMKNGPVQAAFIT-YDDFSGYKGGI
AcCatB          KMCQRKYKNSYKEDKHFAKHSFYLPN-NETSIRQEIFETGPVVAAFNV-YEDFRYYVGGI
HcCBP1          KRCHPGYRKLYRMDKFYGKGAYELPN-SEKAIQREIMKHGPVVGMFRV-FRDFYNYKSGV
HcCBP2          RRCQKGYKKSYKKDRTYGEDAYELPN-SVKAIQKEIMENGPVVAVFTV-YEDFSYYKKGI
HsCatB          KICEPGYSPTYKQDKHYGYNSYSVSN-SEKDIMAEIYKNGPVEGAFSV-YSDFLLYKSGV
HsCatL          ESCKYNPKYSVAND----TGFVDIPK-QEKALMKAVATVGPISVAIDAGHESFLFYKEGI
AcCatL          MKCHFKKKDIGAVD----NGYVDLPEGDEEALKIAVATQGPISIAIDAGHRTFQLYKKGV
                  *.         *         : : .   :   :   **:   :   .  *  *  *:

HcCP1a          YIHTAGA-MEGGHSIKIIGWGVDKGVK----YWLIANSWSTDWGEDGGYFRVVRGINNCD
HcCP1b          YIHTAGK-MKGGHSVKIIGWGVENGTK----YWTIANSWSEDWGENGGYFRVVRGIDNCY
HcCP1c          YVHVKGR-ERGAHAVKLIGWGVENGTK----YWTVANSWHDDWGENG-FFRILRGVNHCE
AcCatB          YVHKWGK-QTGAHAVKVIGWGTENGTD----YWLVSNSWSSDWGEKG-YFRIVRGINNCG
HcCBP1          YKHTAGS-QEGEHAVKIIGWGKEGETP----YWLIANSWHHDWGENG-FFKMLRGSNHCR
HcCBP2          YVHIAGK-ARGAHAIKIIGWGVENGLP----YWLIANSWHDDWGEQG-LFRIVRGINECG
HsCatB          YQHVTGE-MMGGHAIRILGWGVENGTP----YWLVANSWNTDWGDNG-FFKILRGQDHCG
HsCatL          YFEPDCSSEDMDHGVLVVGYGFESTESDNNKYWLVKNSWGEEWGMGGYVKMAKDRRNHCG
AcCatL          YYDEECSSEELDHGVLLVGYGTDPEAG---DYWLVKNSWGTGWGEKGYIRIARNRNNHCG
                * .         *.: ::*:* :        ** : ***   **  *         :.*

HcCP1a          IEGGVLAGTF---------
HcCP1b          IESGVLAGTF---------
HcCP1c          FESYVVSGEFRI-------
AcCatB          IEEEMVAGLMKS-------
HcCBP1          IEELVVAGLVDNDVTRRL-
HcCBP2          IEGDVVAGHVQG-------
HsCatB          IESEVVAGIPRTDQYWEKI
HsCatL          IASAASYPTV---------
AcCatL          VATKASYPLV---------
                .
```

Supplementary Figure S3. Protein sequences were aligned by ClustalW and the His-His motif of the occluding loop is highlighted. AccatB (RCN47340) and AccatL (AAL37181) are the cathepsins B and L from A. caninum, HscatB (AAH95408) and HscatL (KAI4007581) are the cathepsins B and L from H. sapiens.

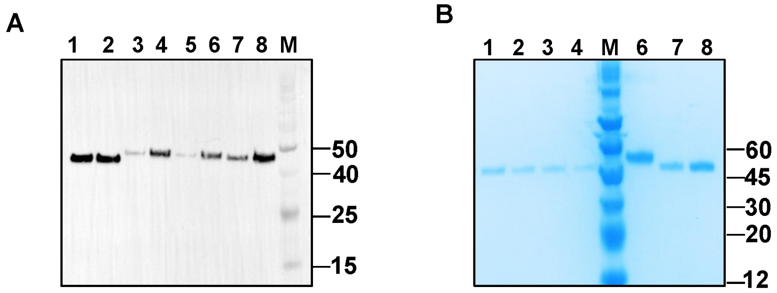

Supplementary Figure S4. Analysis of mHcCPs by Western blotting using the anti-His antibody (A) and SDS-PAGE with Coomassie blue staining (B). A: Lane M, protein molecular standards; Lanes 1 and 2, mHcCP1a from 5-day culture supernatants; Lanes 3 through 6, mHcCP1b from 2-4-day culture supernatants; Lanes 7 and 8, mHcCP1c from 2-4-day culture supernatants. B: Lanes 1-4, purified mHcCP1a; Lane M, molecular standards; Lane 6, mHcCP1c; Lanes 7 and 8, mHcCP1b.

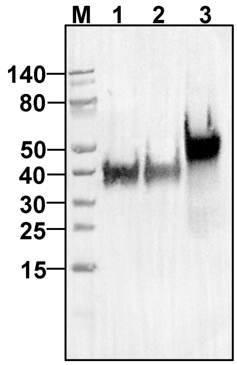

Supplementary Figure S5. Western blot analysis of mHcCBP2 in FreeStyle™ 293-F transfected cell supernatant by using the anti-HcCBP2 antibody. Lane M, protein molecular standards; Lanes 1, 2, and 3, mHcCBP2 collected at different time points in suspension culture.

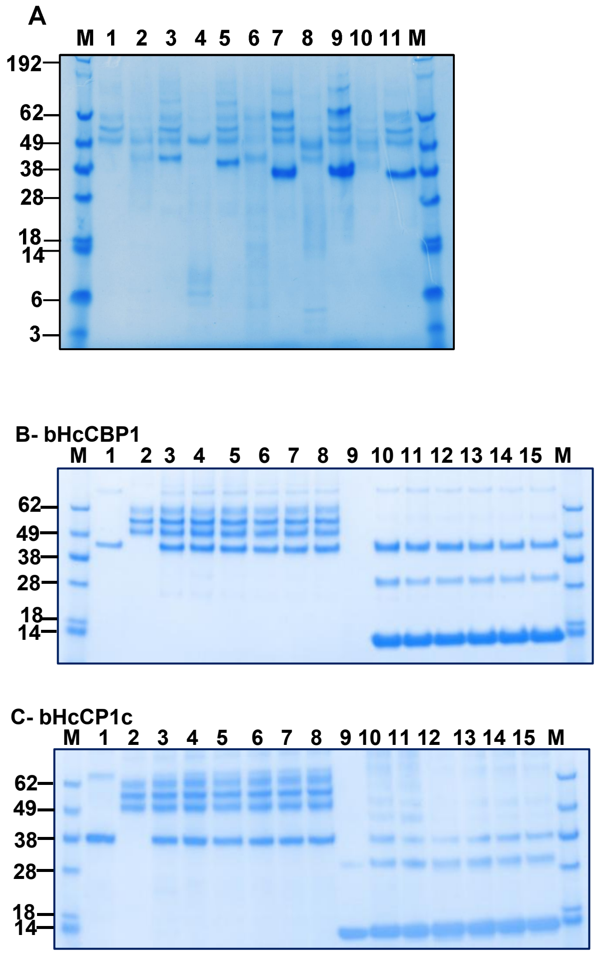

Supplementary Figure S6. SDS-PAGE with Coomassie blue staining of ovine fibrinogen(oFg) digestion by mammalian cell expressed (mHcCPs) as well as *E. coli* expressed *H. contortus* cysteine proteases (bHcCPs). Lane M, protein molecular standards; Lane 1, oFg alone; Lane 2, mHcCBP1 and oFg; Lane 3, bHcCBP1 and oFg; Lane 4, mHcCBP2 and oFg; Lane 5, bHcCBP2 and oFg; Lane 6, mHcCP1a and oFg ; Lane 7, bHcCP1a and oFg ; Lane 8, mHcCP1b and oFg; Lane 9, bHcCP1b and oFg; Lane 10, mHcCP1c and oFg; Lane 11, bHcCP1c and oFg; digestion was performed for 16 h at 37°C in PBS, pH 7.4. B. Cyteine protease activity of bHcCPs on ovine fibrinogen (oFg) and ovine hemoglobin (oHb) at pH 4.0-8.0 at 37°C for 16 h. Proteins were analyzed by SDS-PAGE followed by Coomassie blue staining. (B) bHcCBP1, (C) bHcCP1c, Lanes 3 and 10, 0.1 M acetate buffer (pH 4.0); Lanes 4 and 11, 0.1 M acetate buffer (pH 5.5); Lanes 5 and 12, 0.1 M phosphate buffer (pH 6.5); Lanes 6 and 13, PBS (pH 7.4); Lanes 7 and 14, 0.02 M Tris buffer (pH 8.0); and Lanes 8 and 15, cathepsin B assay buffer (pH 7-8). Lane M, protein standards. Lane 1, HcCPs alone; Lane 2, oFg alone; Lanes 3 through 8, oFg plus HcCPs, Lane 9, oHb alone; Lanes 10 through 15, oHb plus HcCPs B. Lane 9, blank.

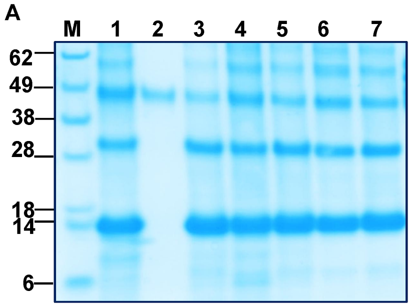

Supplementary Figure S7. SDS-PAGE analysis of blood protein substrate degradation by mHcCBP1 at 37°C for 16 h. Lane M, protein molecular standards; Lanes, 1, 4 and 6, oHb plus mHcCBP1 at pH 4.0 (0.1M acetate buffer), pH 5.0 (0.1M acetate buffer), and pH 6.0 (0.1M phosphate buffer), respectively; Lane 2, mHcCBP1 alone; Lanes 3, 5 and 7, oHb alone at pH 4.0, 5.0 and 6.0, respectively.

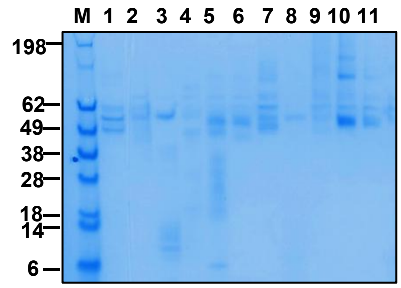

Supplementary Figure S8.The ability of FreeStyle 293-F cells-expressed *H. contortus* cysteine proteases (mHcCPs) to degrade ovine fibrinogen (oFg) at 42°C at pH 7.4 for 16 h. Proteins were analyzed by SDS-PAGE followed by Coomassie blue staining. A. Lanes 1 through 6, pH 7.4 (PBS) without DTT; Lanes 7 through 11, pH 7.4 (PBS) with 10 mM DTT; Lane 1, oFg alone; Lanes 2 and 7, oFg and mHcCBP1; Lanes 3 and 8, oFg and mHcCBP2; Lanes 4 and 9, oFg and mHcCP1a; Lanes 5 and 10, oFg and mHcCP1b; Lanes 6 and 11, oFg and mHcCP1c.

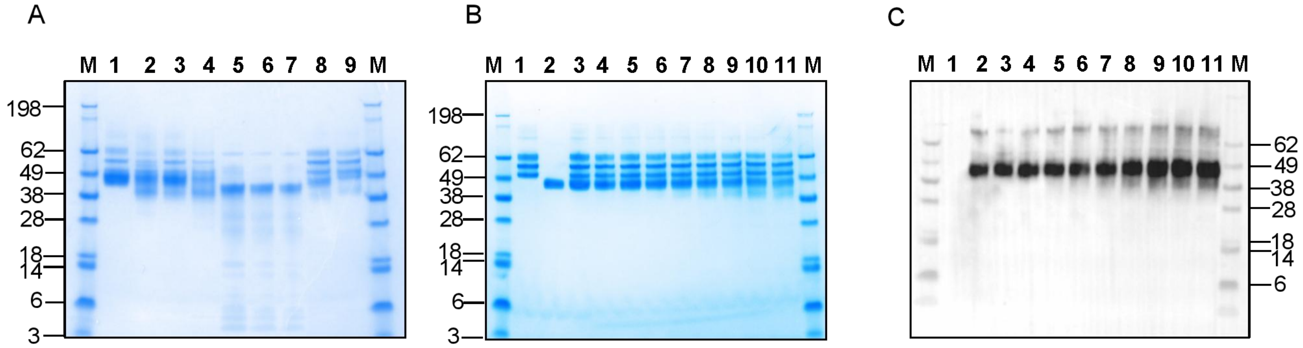

Supplementary Figure S9. SDS-PAGE with Coomassie blue staining (A and B) and Western blot analysis (C) of time-dependent degradation of ovine fibrinogen (oFg) by mHcCBP1 and mHcCP1c at 37°C in PBS, pH 7.4. A. Lane M, protein molecular standards; Lanes 1 through 9, oFg digested by mHcCBP1 and mHcCP1c together for 0 h (Lane 1), 3 h (Lane 2), 6 h (Lane 3), 9 h (Lane 4),12 h (Lane 5), 15 h (Lane 6), and 18 h (Lane 7); Lane 8, mHcCBP1 with oFg for 4 h; Lane 9, mHcCP1c with oFg for 4 h. B and C. Lane M, protein molecular standards; Lanes 1 and 2, oFg and mHcCBP1 alone, respectively; Lanes 3 through 11, oFg digested by mHcCBP1 for 0 h (Lane 3), 1 h (Lane 4), 1.5 h (Lane 5), 2 h (Lane 6), 2.5 h (Lane 7), 3 h (Lane 8), 3.5 h (Lane 9), 4 h (Lane 10), and 4.5 h (Lane 11); Western blot analysis (C) used the anti-HcCBP1 sera.

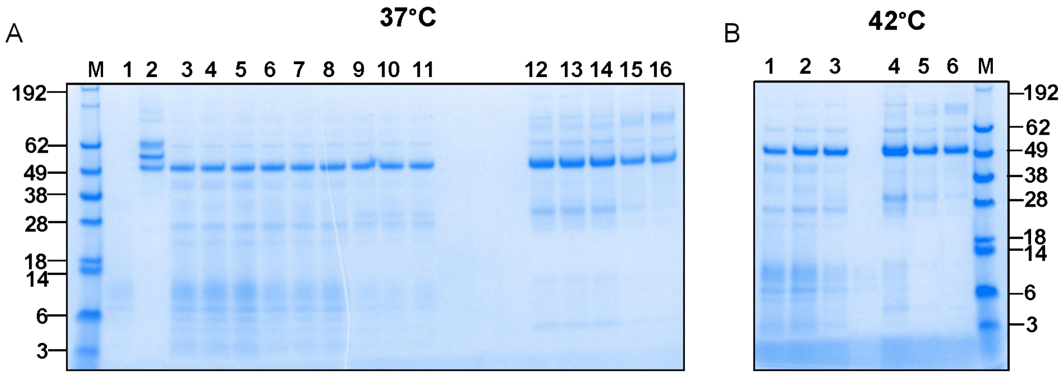

Supplementary Figure S10. Dose-dependent degradation of ovine fibrinogen (oFg) by mHcCBP2 at pH 7.4 in PBS at 37°C (A) and 42°C (B) for 16 h. Proteins were separated by SDS-PAGE and stained by Coomassie blue stain. A. Lane M, protein standards; Lane 1, mHcCBP2 alone; Lane 2, oFg alone; Lanes 3 through 16, constant amount of oFg (2μg) plus mHcCBP2 at 4μg (Lanes 3, 4, and 5), 2μg (Lanes 6, 7, and 8), 1μg (Lanes 9, 10, and 11), 250ng (Lanes 12,13, and 14), 125ng (Lane 15), or 62.5 ng (Lane 16). B. Lane M, protein standards; Lanes 1 through 6, constant amount of oFg (2μg) plus mHcCBP2 at 4μg (Lane 1), 2μg (Lane 2), 1μg (Lane 3), 250ng (Lane 4), 125ng (Lane 5), or 62.5 ng (Lane 6).

A

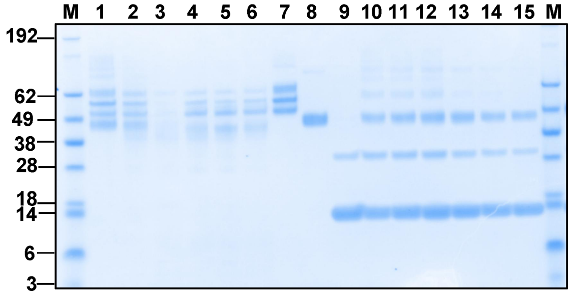

Supplementary Figure S11. SDS-PAGE with Coomassie blue staining of temperature-dependent degradation of oFg (Lanes 1-7) or oHb (Lanes 9-15) by mHcCBP1 in PBS at pH 7.4 for 16 h. Lane M, protein standards; Lanes 1 through 6, mHcCBP1 and oFg; Lane 7, oFg alone; Lane 8, mHcCBP1 alone; Lane 9, oHb alone; Lanes 10 through 15, mHcCBP1 and oHb. Lanes 1 and 10 (48.7°C); Lanes 2 and 11 (46.8°C); Lanes 3 and 12 (44.7°C); Lanes 4 and 13 (42°C); Lanes 5 and 14 (39.9°C); and Lanes 6 and15 (37.7°C).

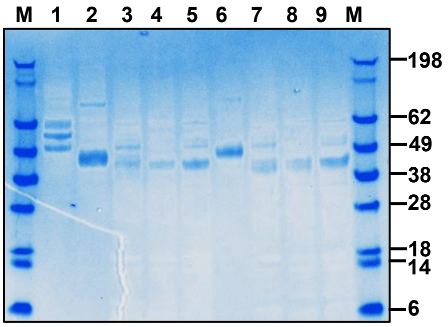

Supplementary Figure S12. SDS-PAGE analysis of enzymatic degradation of oFg by mHcCBP1 and mHcCP1c at 37°C in PBS at pH 7.4. Lane M, protein standards; Lane 1, oFg alone incubated for 24 h at 37°C; Lane 2, mHcCBP1 alone incubated for 24 h at 37°C; Lane 3, mHcCBP1 alone incubated for 12 h before adding oFg followed by additional incubation for 12 h. Lane 4, mHcCBP1 and oFg incubated for 24 h; Lane 5, mHcCBP1 plus oFg incubated for 12 h before adding oFg and continued to incubate for additional 12 h; Lane 6, mHcCP1c alone incubated for 24 h at 37°C; Lane 7, mHcCP1c alone incubated for 12 h before adding oFg and continued to incubate for additional 12 h; Lane 8, mHcCP1c and oFg incubated for 24 h; Lane 9, mHcCP1c plus oFg incubated for 12 h before adding oFg and continued to incubate for additional 12 h.
